# Self-passivated bilayer black phosphorus QDs based multifunctional nanoparticles for tumor immune reprogramming

**DOI:** 10.1016/j.mtbio.2026.102862

**Published:** 2026-02-02

**Authors:** Tingting Liu, Wenyan She, Ruili Du, Yali Bao, Zhibin Guo, Qichao Gao, Hanping Li, Pengfei Suo, Yi Liu, Yujiao Liu

**Affiliations:** a State Key Laboratory of Advanced Separation Membrane Materials, School of Chemistry & School of Material Science and Engineering & School of Chemical Engineering and Technology, Tiangong University, Tianjin, 300387, PR China; b School of Pharmaceutical Sciences, Key Laboratory of Targeting Therapy and Diagnosis for Critical Diseases, Zhengzhou University, Zhengzhou, 450001, PR China; c Technology Department, Long Xun Kuang Teng Inc. Beijing, Beijing, 100192, PR China; d School of Chemical and Environmental Engineering, Wuhan Polytechnic University, Wuhan, 430023, PR China

**Keywords:** Immunotherapy, PD1, Black phosphorus quantum dots, Photodynamic therapy, Density functional theory

## Abstract

It is important yet challenging to enhance immunotherapy responses using biosafe agents due to the immunosuppressive tumor microenvironment. To address this challenge, BD3PP was constructed by encapsulating black phosphorus quantum dots (BPQDs), a synthesized thioredoxin reductase inhibitor 3c, and Dir (the fluorescent dye) into PLGA nanoparticles, followed by conjugation with a PDL1 antagonist for synergistic multimodal therapy and imaging. The mechanism and efficiency of BD3PP were investigated through density functional theory (DFT) calculations, molecular docking, and *in vitro* and *in vivo* experiments. The PDL1 antagonist served as a targeting moiety that binds PDL1 on the tumor cell surface, enabling the controlled intracellular release of the three therapeutic agents. Self-passivated bilayer BPQDs converted optical energy into heat for photothermal therapy and generated singlet oxygen (^1^O_2_) from O_2_ for type II photodynamic therapy, showing far superior to non-passivated bilayer BPQDs or bulk BP. Meanwhile, **3c** selectively inhibited thioredoxin reductase, leading to the production of **·**$O_{2}^{‐}$ and H_2_O_2_. These effects synergistically induced immunogenic cell death (ICD), promoted macrophage polarization toward the M1 phenotype, and remodeled the tumor microenvironment to facilitate tumor clearance. The near-infrared fluorescent dye Dir enabled real-time imaging both *in vitro* and *in vivo*. DFT calculation revealed that BPQDs were ultimately degraded into biocompatible phosphoric acid. Along with the other biocompatible components in BD3PP, biosafety was guaranteed. This research introduces an efficient and biosafe nanoplatform based on self-passivated bilayer BPQDs, which exhibits prolonged blood circulation and enhanced multimodal real-time photothermal and near-infrared imaging. Importantly, this nanoplatform enables integrated photothermal, photodynamic, and targeted therapies, demonstrating promising potential for anti-tumor preclinical and clinical applications.

## Highlights

• Ultrasmall, homogeneous, self-passivated bilayer BPQDs were successfully prepared.
• The PDT mechanism and the biodegradation of BPQDs were firstly investigated by DFT.
• BD3PP induced ICD to remodel the tumor microenvironment to clear tumors.
• BD3PP prolonged blood circulation, multimode imaging and therapy efficiency.

## Graphical abstract

**Figure:**
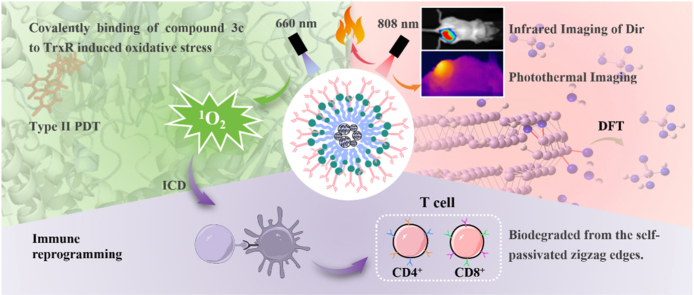

## 1 Introduction

Immunotherapy, particularly immune checkpoint blockade (ICB) strategies, has shown considerable promise in the treatment of various cancer types [[1], [2], [3]]. However, the immunosuppressive tumor microenvironment (TME) severely restricts the efficacy of immunotherapy against solid tumors, including triple-negative breast cancer (TNBC) [4,5]. The infiltration of immunosuppressive cells, particularly tumor-associated macrophages (TAMs), plays a major role in the upregulation of immune checkpoints and the functional exhaustion of T cells [6].

TAMs constitute a heterogeneous population, comprising anti-inflammatory and pro-tumorigenic M2-like TAMs, as well as pro-inflammatory and tumor-suppressive M1-like TAMs. Clinical studies have demonstrated that elevated levels of TAMs, particularly those with an M2-like phenotype, are frequently associated with poorer patient prognoses [7,8]. Moreover, recent studies underscore the pivotal role of TAMs in mediating therapy resistance and tumor progression through mechanisms such as impaired antigen presentation, enhanced angiogenesis, and extracellular matrix remodeling [[9], [10], [11]]. Therefore, inducing the polarization of TAMs toward the M1 phenotype represents a promising strategy to remodel the TME and potentiate the efficacy of cancer immunotherapy [12].

However, the efficacy of ICB monotherapy is limited by multiple mechanisms within the TME. Only approximately 20–40% of patients respond to ICB therapy, and even fewer achieve long-term disease remission. To overcome these limitations, four strategies have been proposed: targeting effector T cells [13]; modulating the TME and stromal cell [14,15]; innate immune and regulatory cells; and cancer cells. These approaches aim to provide synergistic clinical benefits when combined with PD1 axis blockade [16,17].

A multivalent immune checkpoint therapeutic platform (Nb-Ftn@ICG) was developed by conjugating anti-PDL1 nanobodies (Nb) to ferritin nanocages (Ftn). Nb-Ftn@ICG induces immunogenic death (ICD) of tumor cells by promoting dendritic cell maturation and enhancing T cell infiltration, thereby ablating primary tumors, suppressing abscopal tumors, and inhibiting tumor metastasis [18]. In addition, nano Z-scheme heterojunctions Mn_*x*_O_*y*_/(A/R) TiO_2_ (MTO), in combination with PD-L1 checkpoint blockade, have been shown to induce robust systemic immunity and durable immune memory, thereby inhibiting tumor progression, metastasis, and recurrence through enhancing ICD [19].

Photothermal materials have been widely explored in antitumor therapy owing to their ability to efficiently convert light energy into localized heat, enabling minimally invasive and spatially controllable tumor ablation [[20], [21], [22]]. Among various nanomaterials, black phosphorus (BP) stands out as a promising two-dimensional biodegradable material with a large specific surface area and high chemical reactivity [23]. Its puckered layered structure is primarily derived from the sp^3^ hybridization of P atoms. The presence of lone pair electrons renders BP chemically reactive. The biodegradation of nano BP in the presence of O_2_ and H_2_O disrupts intracellular ion homeostasis and redox balance, thereby inhibiting tumor growth [24]. In addition to its efficient drug delivery capabilities and excellent biocompatibility, nano-BP can function as a photothermal core. Moreover, it can serve as a photosensitizer for tumor photothermal and photodynamic therapy (PTT/PDT) by absorbing optical energy to generate heat or ^1^O_2_ [25].

**Scheme 1:**
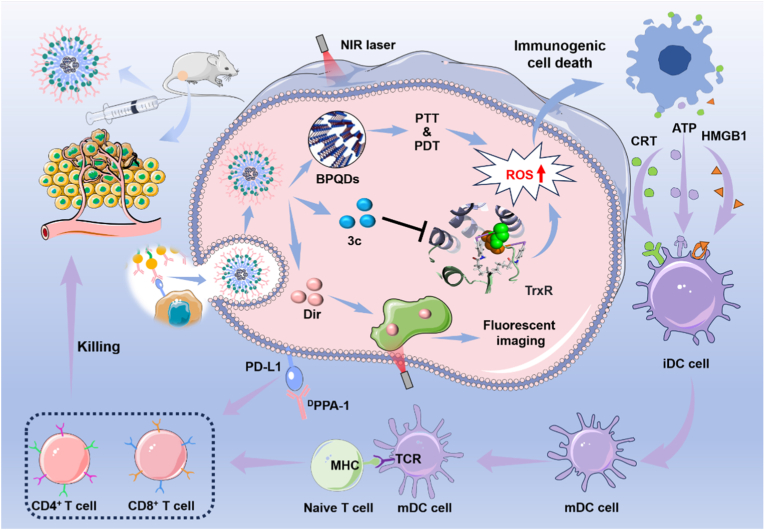
Schematic illustration of the therapeutic mechanism of BD3PP, which is composed of black phosphorus quantum dots (BPQDs), the thioredoxin reductase inhibitor **3c**, the fluorescent dye Dir, and the PDL1 antagonist ^D^PPA-1. In combination with PD1 axis blockade, BD3PP reprograms the immunosuppressive TME through photothermal, photodynamic, and targeted therapies, assisted by photothermal and near infrared imaging.

In this study, a multifunctional photoimmune targeting nanoplatform was developed. Photothermal core (self-passivated bilayer BPQDs) was prepared via liquid exfoliation of BP, serving as both a delivery scaffold and a photosensitizer for PDT and PTT (Scheme 1). In combination with the ^1^O_2_ generated by the photosensitizer core, the synthesized TrxR-targeted inhibitor **3c** was introduced to produce additional reactive oxygen species (ROS), such as superoxide (**·** $O_{2}^{‐}$) and hydrogen peroxide (H_2_O_2_), thereby synergistically inducing ICD and activating antitumor immunity. The fluorescent tracer Dir enabled near-infrared (NIR) visualization of the nanoplatform both *in vitro* and *in vivo*. The photosensitizer core, TrxR-targeted inhibitor, and fluorescent tracer were encapsulated within PLGA polymers to form the multifunctional photoimmune targeting nanoplatform. To facilitate targeted delivery to tumors, the immune converter (PDL1 antagonist ^D^PPA-1) was conjugated to the surface of the PLGA-encapsulated nanoparticles. This multifunctional nanoplatform enables targeted delivery of these theranostic agents and reprograms the immunosuppressive TME to achieve multimodal antitumor therapy.

## 2 Results and discussion

### 2.1 Preparation and characterization of multifunctional photoimmune targeting nanoplatforms (BD3PP)

**Fig. 1:**
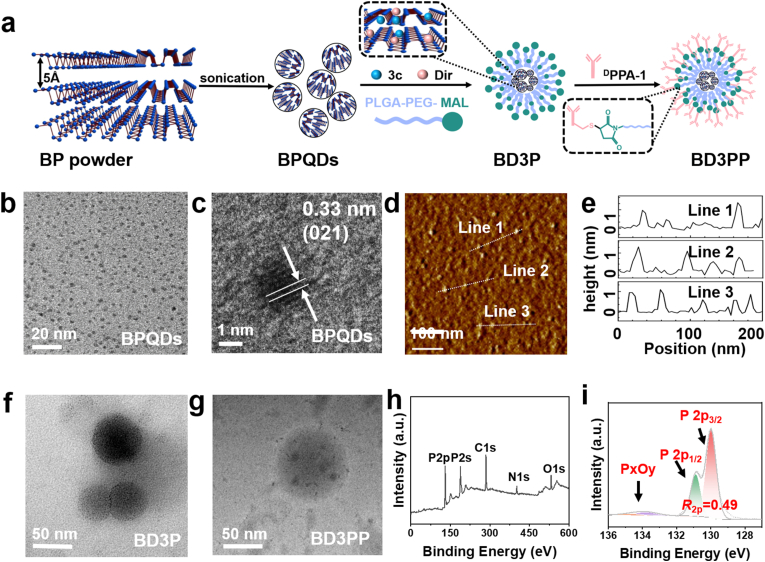
Preparation and characterization of multifunctional photoimmune targeting nanoplatforms (BD3PP). (a)Schematic illustration of the preparation of BD3PP. (b, c) TEM image and high-resolution TEM image of BPQDs, respectively. (d, e) AFM image and corresponding cross-sectional analysis of the size and height of BPQDs. (f, g) TEM images of BD3P and BD3PP, respectively. (h, i) XPS spectra of self-passivated BPQDs.

Probe sonication combined with bath sonication was employed to exfoliate bulk black phosphorus crystals into uniform and ultrasmall BPQDs (**b**lack **p**hosphorus **q**uantum **d**ots, Fig. 1a). The average size of the BPQDs was approximately 2.3 nm, as determined from TEM images (Fig. 1b). High-resolution TEM image (Fig. 1c) revealed lattice fringes with a spacing of 0.33 nm, which can be attributed to the (021) plane of the BP crystal [26]. The other lattice planes of BPQDs are presented in Fig. S1a and S1b. Atomic force microscopy (AFM) images (Fig. 1d) displayed the topographic morphology of the BPQDs, and the thickness was determined by cross-sectional analysis. The measured height of 1.3 nm corresponded to BPQDs consisting of 2 layers (Fig. 1e). Collectively, these data demonstrate that uniform and ultrasmall bilayer BPQDs were successfully prepared.

Owing to the large interlayer spacing of BPQDs, various cargos, such as targeting molecules, can be readily loaded into BPQDs. Furthermore, considering the poor stability of BPQDs in O_2_-containing environments, surface coating strategies should be employed to prevent degradation. In this study, the TrxR targeting molecule **3c** and the fluorescent dye Dir were encapsulated within PLGA-PEG-MAL using an oil-in-water emulsion solvent evaporation method, resulting in the formation of BD3P (3: molecule **3c**; P: **P**LGA-PEG-MAL). This encapsulation strategy enabled the sustained release of therapeutic payloads. The MAL (maleimide) -terminalized polymer provided surface binding sites for the PD-L1 antagonist ^D^PPA-1 via a reaction between the thiol group of ^D^PPA-1 and the maleimide moiety, resulting in the formation of BD3PP (the final P: ^D^**P**PA-1).

As demonstrated by TEM images (Fig. 1f and g), both BD3P and BD3PP exhibited regular spherical morphologies, good dispersibility, and significantly larger sizes compared to BPQDs. Dynamic light scattering (DLS) analysis revealed that the average hydrodynamic diameters (Fig. 1h) of BPQDs, BD3P, and BD3PP were 7.5 nm, 65.5 nm, and 78.2 nm, respectively. All nanoparticles exhibited negative surface charges (Fig. S1). Elemental analysis of BD3PP (Fig. 1i) confirmed the simultaneous presence of P (from BPQDs), S (from PD-L1 antagonist ^D^PPA-1 and **3c**) and As(from **3c**), indicating the successful encapsulation of all components within BD3PP. The release profiles of 3c from BD3PP at different pH demonstrate pronounced pH-responsive behavior, with the highest release rate observed under acidic conditions, moderate release at neutral pH, and the lowest release rate in alkaline environments (Fig. S1i).

The TrxR-targeted drug **3c** was efficiently incorporated into BD3PP nanoparticles, as determined by HPLC analysis, achieving a drug loading efficiency of 14.2 % and an encapsulation efficiency of 78.0 % (Table 1). Consistent results were further obtained by inductively coupled plasma (ICP) analysis (Table S1). In addition, no free ^D^PPA-1 (^D^PPA-1 lane) was detected in the BD3PP lane. All ^D^PPA-1 in the BD3PP lane remained stationary in the gel stained with Coomassie Brilliant Blue, confirming the successful incorporation of ^D^PPA-1 into BD3PP (Fig. S2).

**Table 1:** Drug loading efficiency and encapsulation efficiency determined by HPLC.

	Sample 1	Sample 2	Sample 3	mean ± SD
Dry BD3PP weight/mg	5.4	5.9	5.3	
3c weight/mg	0.786	0.795	0.760	
Loading efficiency/%	14.6	13.5	14.4	14.2 ± 0.5
Encapsulation efficiency/%	78.6	79.5	76.0	78.0 ± 1.8

X-ray photoelectron spectroscopy (XPS) was employed to investigate the surface chemical states of the self-passivated BPQDs (Fig. 1h and i). As shown in Fig. 1h, a weak O 1s peak at 532.1 eV and a very low proportion of phosphorus oxides (Fig. 1i) indicated a low oxidation degree and minimal oxygen-related passivation. The high-resolution P 2p spectrum (Fig. 1i) exhibited two characteristic peaks at 130.1 eV and 131.3 eV, corresponding to P 2p_3/2_ and P 2p_1/2_, respectively. The calculated area ratio between P 2p_1/2_ and P 2p_3/2_ (*R*_2p_) was 0.49, close to the theoretical value of 0.5, suggesting a relatively intact and self-passivated BP structure. These results provided direct experimental evidence for the self-passivation and edge reconstruction of bilayer BPQDs.

### 2.2 Photothermal and photodynamic performance

**Fig. 2:**
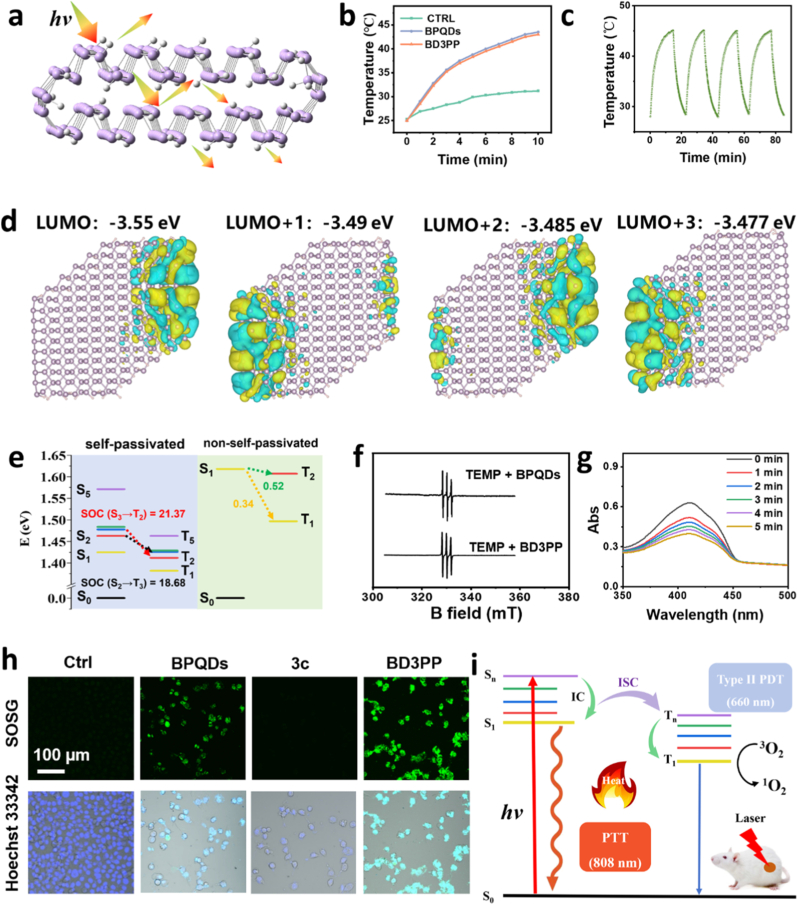
Photothermal and photodynamic performance. (a) Schematic illustration of heat generation resulting from the interaction between photons and BPQDs. (b) Temperature curves of water with/without BPQDs and BD3PP. (c) Temperature change curves of BD3PP during 4 cycles of 808 nm laser irradiation (1.0 W/cm^2^) (d) Molecular orbitals of BPQDs with self-passivation. (e) Energy levels of singlet and triplet states of BPQDs with self-passivation (left) and without self-passivation (right). (f) ESR spectra of BPQDs and BD3PP after 5 min of 660 nm laser irradiation, using TEMP as the spin-trapping agent for ^1^O_2_. (g) Time-course absorption spectra of DPBF solution treated with BPODs and irradiated with 660 nm light at a power density of 80 mW/cm^2^. (h) Confocal images of 4T1 cells treated with of BPQDs, **3c,** and BD3PP, probed by SOSG. Green: SOSG for ^1^O_2_ Blue: Hoechst 33342 for cell nuclei. (i) Modified Jablonski diagram illustrating the PTT and PDT mechanisms of BPQDs.

Micro/nanostructured materials, for example BPQDs, gain incident photo energy through trapping photos in the nanostructures by increasing the times of reflections, refractions, and scatterings (Fig. 2a). The absorbed photon energy is released into the environment by heat radiation, contributing to photothermal effects. As depicted in Fig. 2b–S2b, after 10 min of irradiation with an 808 nm laser, both the BPQDs and BD3PP showed a rapid and significant increase from ambient temperature to a maximum of ∼45 °C, sufficient heat to induce tumoral apoptosis. In comparison, the temperature of the PBS only increased to 31 °C. Moreover, after multiple photothermal cycles, the photothermal performance of BD3PP remained stable (Fig. 2c). The photothermal conversion efficiency of BD3PP was 25.2%.

Density functional theory (DFT) and time-dependent density functional theory (TD-DFT) calculations were also conducted to investigate the photodynamic properties of BPQDs. The edge configuration plays a crucial role in determining the physical and chemical properties of low-dimensional materials. During the preparation process, BP flakes typically generate zigzag, armchair, and skewed diagonal pristine edges [27]. Furthermore, the zigzag edge of bilayer BP undergoes reconstruction, resulting in self-passivated edge structures [28]. Based on the characterization of BPQDs in Fig. 1, two bilayer quantum dot models (∼2.3 nm), model 1 and model 2, featuring armchair, skewed diagonal, and zigzag edges (Fig. S3), were constructed to explore the interaction between photons and BPQDs. In model 2, the zigzag edge of bilayer BPQDs formed self-passivated edge structures, which did not occur in model 1.

In model 1, the highest occupied molecular orbital (HOMO) energy level was calculated to be −5.10 eV, while the lowest unoccupied molecular orbital (LUMO) energy level was −3.12 eV (Fig. S4). The energy gap between the HOMO and LUMO, a critical parameter for determining electronic properties, was 1.98 eV, which is significantly larger than that of bulk bilayer black phosphorous (∼1 eV), likely due to quantum confinement effect. In real space, based on the state distribution and the anti-bonding and bonding features (Fig. S4, red frame), the HOMO and LUMO states corresponded well to the valence band maximum (VBM) and conduction band minimum (CBM), respectively, of bilayer black phosphorus (Fig. S5).

The lowest singlet excited state (S_1_) of model 1 was a bright state with an energy of 1.641 eV, corresponding to an absorption wavelength of 756 nm, which falls within the recommended range for photosensitizers to achieve effective tissue penetration (600–850 nm). However, the spin-orbit coupling (SOC) between S_1_ and T_1_ and between S_1_ and T_2_ were very low (0.34 and 0.52 cm^−1^, respectively, Fig. 2e), indicating that intersystem crossing from the singlet to the triplet state is inefficient and thus not favorable fo^1^O_2_ generation under near-infrared light irradiation.

Compared with model 1, the energies of the higher excited states (S_n_ and T_n_) of self-passivated bilayer BPQDs (model 2) gradually decreased and approached the ground state. The HOMO state was largely unaffected by the self-passivated boundary, whereas the LUMO state was significantly influenced, where LUMO, LUMO+1, LUMO+2, and LUMO+3 were all boundary states arising from the self-passivated edge (Fig. 2d and S6). The four lowest-energy singlet excited states (S_1_ to S_4_) were dark states, which are quantum system that cannot be accessed via photon absorption. S_5_ (corresponding to the transition from HOMO to LUMO+4) was a bright state with an energy at 1.571 eV, corresponding to an absorption wavelength of 790 nm, which also satisfies the absorption requirements for photosensitizers.

Although the SOC values (Table 2) between the bright state S_5_ and the triplet states (T_n_) were weak, all four dark states exhibited strong SOC with the triplet states, particularly S_2_ to T_3_ (18.68 cm^−1^) and S_3_ to T_2_ (21.37 cm^−1^). Therefore, following near-infrared light excitation to the bright state, electrons can first transit to the dark states and subsequently to the triplet states, thereby facilitating ^1^O_2_ generation. Moreover, the S_0_ to T_1_ energy gap (1.382 eV) was greater than the energy required for ^1^O_2_ formation ^1^O_2_ (0.98 eV) or the experimentally determined value in solution (1.13 eV) [29].

**Table 2:** SOC values between S_n_ and T_n_ states of self-passivated BPQDs.

SOC (cm^−1^)	T1	T2	T3	T4	T5
S1	2.68	1.44	12.37	20.37	0.53
S2	0.73	12.38	18.68	10.65	2.18
S3	1.93	21.37	9.62	7.60	1.73
S4	23.64	2.63	1.73	2.23	1.17
S5	0.82	1.29	0.62	1.18	0.65

Indeed, a characteristic signal resulting from the conversion of TEMP to 2,2,6,6-tetramethylpiperidine-1-oxyl (TEMPO) by ^1^O_2_ was observed using electron spin resonance (ESR) spectroscopy (Fig. 2f). Additionally, the decreased absorbance intensity of the probe DPBF (1,3-Diphenylisobenzofuran, Fig. 2g) with increasing incubation time further confirmed the generation of ^1^O_2_ by BPQDs under NIR laser irradiation. To evaluate the photodynamic therapy (PDT) efficiency, we measured the levels of reactive oxygen species (ROS) generated by BP, non-self-passivated BPQDs, and self-passivated bilayer BPQDs under 660 nm laser irradiation using DCFH-DA as a probe (Fig. S7a, S7b). The results indicated that passivated bilayer BPQDs exhibited superior PDT efficiency, which was consistent with our theoretical studies. Upon addition of NaN_3_, a well-known singlet oxygen scavenger, the fluorescence intensity of all samples sharply decreased to near zero, confirming the involvement of ^1^O_2_ in the fluorescence process and validating the effectiveness of the PDT mechanism. Furthermore, under BPQDs or BD3PP treatment and NIR light irradiation, a bright green fluorescent signal was detected in both tubes and 4T1 cells (Fig. 2h–S7c), further supporting the generation of ^1^O_2_. Moreover, no H_2_O_2_ was detected in the BPQDs or BD3PP groups (Fig. S7d, S7e), indicating a non-type Ⅰ photodynamic therapeutic effect.

Taken together, the PTT and PDT mechanisms of BPQDs are illustrated in Fig. 2i. Upon excitation of BPQDs, there are two main pathways for energy dissipation. The first pathway is vibrational relaxation, a non-radiative process that releases energy as heat, increasing the ambient temperature to approximately 45 °C, thereby enabling effective PTT. The second pathway involves relaxation from T_1_ to S_0_, during which ground-state O_2_ (^3^O_2_) is converted to ^1^O_2_ via ISC (intersystem crossing) from S_n_ with very large SOC values (over 20 cm^−1^), thereby supporting efficient PDT.

### 2.3 Covalent modification of TrxR by organoarsenic 3c suppress thioredoxin redox function

TrxR serves as a crucial redox modulator and is recognized as an important therapeutic target in various cancers, including breast cancer, leukemia, and lung cancer [30]. TrxR is a homodimeric protein, with each monomer containing an active catalytic site composed of a unique Sec residue and an adjacent Cys residue (Sec^498^/Cys^497^) at the flexible C-terminus. This site reduces a wide range of substrates through a selenolthiol/selenenylsulfide exchange reaction.

**Fig. 3:**
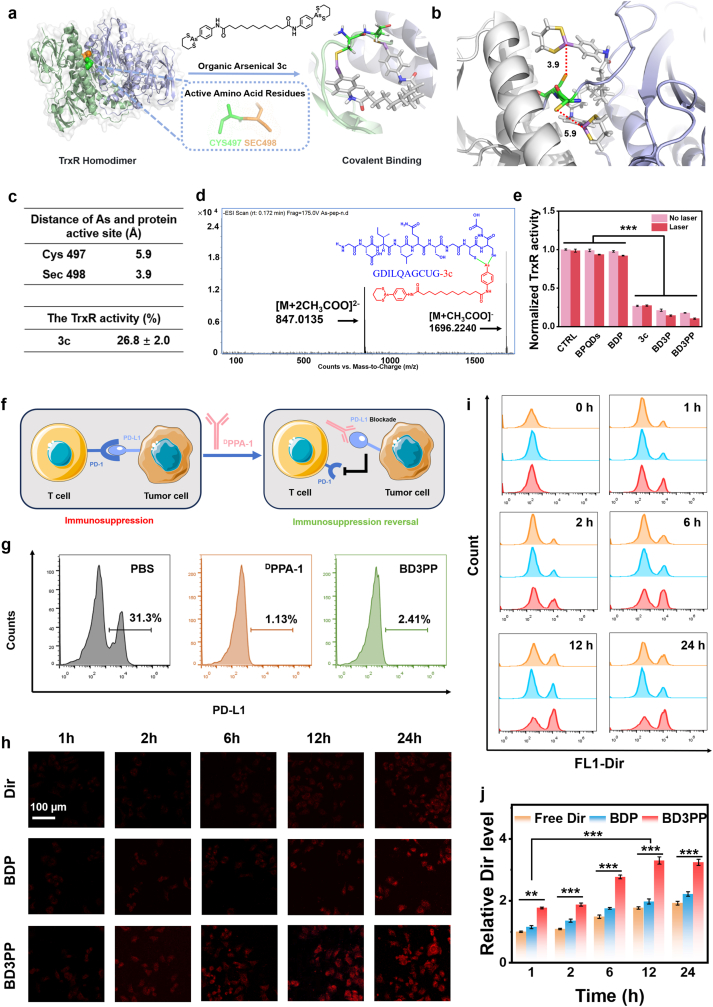
PD-L1 blockade and cellular uptake behavior of BD3PP. (a) Schematic illustration of the covalent interaction between **3c** and TrxR. (b) Molecular docking results of **3c** with TrxR (PDB: 3EAN). (c) Top: Distance between arsenic atom of **3c** and the active sites of the protein. Bottom: Activity of isolated recombinant TrxR incubated with **3C**. (d) MS spectrum of the mixture of the peptide GDILQAGCUG and **3c**. (e) Analysis of TrxR activity in 4T1 cells under different treatments. (f) Schematic illustration of the effect of ^D^PPA-1 on the interaction between PD-1 and PD-L1. (g) Flow cytometric analysis of the PD-L1 blockade effect of PBS, free ^D^PPA-1, and BD3PP on 4T1 cells. (h, i) Cellular fluorescence changes of DIR in 4T1 cells after 1, 2, 6, 12, and 24 h of incubation with free DIR, BD3P, or BD3PP, as observed by confocal microscopy and flow cytometry. (j) Relative DIR levels in 4T1 cells measured by flow cytometry after various treatments.

To elucidate the binding potential of the synthesized compound **3c** with TrxR, molecular docking studies were performed (Fig. 3a). Fig. 3b and S9 show the detailed interactions between compound **3c** and TrxR (PDB:3EAN). The distance between arsenic atom and -SeH of Sec^497^ was 3.9 Å (Fig. 3c), indicating that **3c** can bind to regions near the active site (Cys^497^Sec^498^) of TrxR and form a stable cyclic complex. To confirm the covalent binding, **3c** (*m*/*z* = 775.0312) was mixed with a peptide corresponding to the TrxR C-terminus (GDILQAGCUG, *m*/*z* = 999.32). As shown in Fig. 3d, the peaks at *m*/*z* = 1696.2240 ([GDILQAGCUG-**3c** + CH_3_COO]^-^ and *m*/*z* = 847.0135 ([GDILQAGCUG-**3c** + 2CH_3_COO]^2-^) provide direct evidence for the covalent binding between **3c** and TrxR.

The covalent binding of compound **3c** to TrxR constitutes a key mechanism underlying its functional efficacy. When **3c** was incubated with recombinant TrxR protein at equimolar concentrations, the enzymatic activity of TrxR was reduced to 26.8% of the control (Fig. 3c). In addition, a pronounced suppression of TrxR activity was observed in 4T1 tumor cells (Fig. 3e). These results suggest that **3c** functions as a selective and potent inhibitor of TrxR, thereby contributing to its anti-tumor properties.

Inhibition of TrxR may lead to the production of ROS, such as superoxide (**·** $O_{2}^{‐}$) and hydrogen peroxide (H_2_O_2_). In this study, the H_2_O_2_ content was measured (Fig. S10). BPQDs did not affect H_2_O_2_ production, whereas treatment with **3c** induced a high level of H_2_O_2_, as did BD3PP containing an equivalent amount of **3c**.

### 2.4 PD-L1 blockade and cellular uptake of BD3PP

Overexpression of PD-L1 on the surface of tumor cells typically functions to prevent autoimmunity by engaging with the programmed PD-1 receptor on T cells. Blocking these immune checkpoints with exogenously administered antagonists can disrupt immunosuppressive pathways, thereby unleashing and enhancing pre-existing anti-cancer immune responses by T cells. Studies have shown that ^D^PPA-1 acts as an antagonist of PD-L1 (Fig. 3f) [31]. In this study, free ^D^PPA-1 and PBS were used as positive and negative controls, respectively, and the PD-L1 blockade efficiency of BD3PP on 4T1 cells was assessed by flow cytometry. The initial PD-L1 positivity rate on the surface of 4T1 cells was approximately 31.3% (Fig. 3g). Upon treatment with free ^D^PPA-1 (5 μg/mL), the PD-L1 positivity rate sharply decreased to 1.13%. When 4T1 cells were co-incubation with BD3PP (containing an equivalent concentration of ^D^PPA-1), the PD-L1 positivity rate was 2.41%, indicating that BD3PP can effectively block PD-L1 on tumor cells. These results also demonstrated the successful covalent attachment of ^D^PPA-1 to the nanoparticle surface.

Furthermore, the uptake of BD3PP by 4T1 cells was investigated using laser confocal microscopy and flow cytometry (Fig. 3h–j). Notably, BD3PP exhibited a significantly faster internalization process, with a 1.67-fold higher fluorescence intensity after 12 h of incubation compared to nanoparticles lacking the PD-L1 binding peptide. These findings suggest that the surface-coupled PD-L1 binding peptide confers targeting capability to the nanoparticles, thereby facilitating their uptake and accumulation of drugs within tumor cells. After surface modification with ^D^PPA-1, the nanoparticles exhibited enhanced targeting capability by selectively binding to PD-L1, which is highly expressed on tumor cell membranes. This modification facilitated cellular internalization and improved the efficiency of therapeutic agent delivery. Additionally, ^D^PPA-1 interfered with the interaction between PD-L1 and T cells, thereby alleviating immune suppression and restoring the anti-tumor activity of immune cells.

### 2.5 *In vitro* anti-tumor effects of BD3PP

**Fig. 4:**
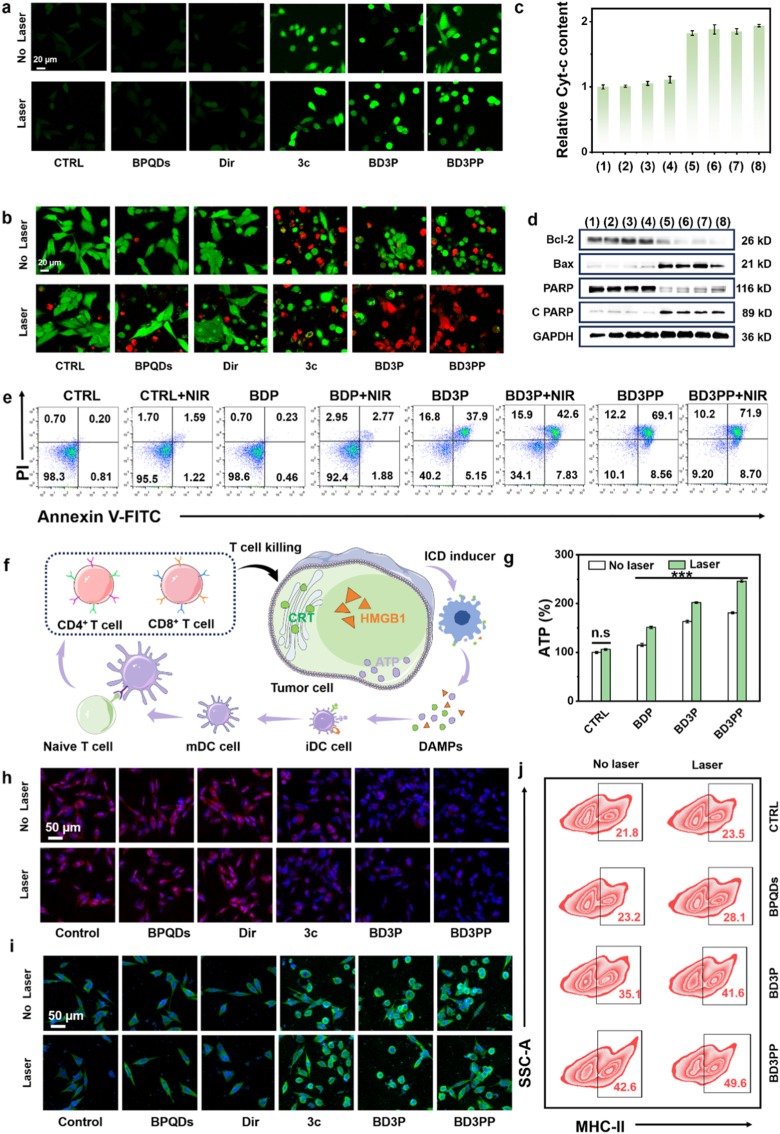
*In vitro* antitumor effects and ICD induced by BD3PP *in vitro*. (a) Confocal fluorescence images of ROS in 4T1 cells after 12 h of treated. ROS probe: DCFH-DA. (b) Fluorescence images of 4T1 cells co-stained with calcein AM (live cells, green fluorescence) and PI (dead cells, red fluorescence). (c) Relative cytochrome *c* levels in 4T1 cells. (1): Control; (2): NIR; (3): BDP; (4): BDP + NIR; (5): BD3P; (6): BD3P + NIR; (7): BD3PP; (8): BD3PP + NIR. (d) western blotting images of apoptosis-related proteins. (e) Flow cytometry analysis of tumor cell apoptosis under different treatments. (f) Schematic illustration of potent systemic anti-tumor immunity activated by ICD. (g) Percentage of extracellular ATP in 4T1cells co-incubated with the material under laser stimulation. Representative confocal fluorescence images of 4T1 cells from different groups: (h) HMGB1, red fluorescence; (i) CRT, green fluorescence; nucleus, blue fluorescence. (j) flow cytometry analysis of MHC-II expression on the surface of mature DCs.

BD3PP induced the generation of ^1^O_2_ under NIR light irradiation (Fig. 2h), while compound **3c** promoted the production of ROS, such as H_2_O_2_, through TrxR inhibition (Fig. S10). In combination with targeted therapy and photothermal treatment, BD3PP induced severe oxidative stress (Fig. 4a, S11), as demonstrated by confocal imaging and flow cytometry (Fig. S11B). In contrast, cells in the CTRL (control), BPQDs, and Dir groups exhibited minimal ROS levels with/without laser irradiation, thereby maintaining cell viability (Fig. S12). Significant tumor cell death was observed following incubation with **3c**, BD3P, or BD3PP, as indicated by the fluorescence (Fig. 4b) of the cells co-stained with calcein AM (live cells, green fluorescence) and propidium iodide (PI, dead cells, red fluorescence).

Furthermore, Western blotting analysis (Fig. 4c) revealed upregulation of BAX and downregulation of Bcl-2 expression in the BD3P and BD3PP groups, indicating activation of the mitochondrial apoptotic pathway. In addition, cytochrome *c* was released into the cytosol (Fig. 4d) to initiate apoptosis, as evidenced by PARP activation. Annexin V-FITC and PI double staining experiments (Fig. 4e–S13) provided further evidence that cell death occurred via apoptosis and that the components of BD3PP acted synergistically.

### 2.6 BD3PP-induced ICD *in vitro*

ROS have the capacity to directly kill tumor cells and induce ICD within these cells [[32], [33], [34]]. During ICD, adenosine triphosphate (ATP) is actively exported from the intracellular space, serving as a “find-me” signal that potently attracts immune cells to the site of dying cells (Fig. 4f). As shown in Fig. 4g, BD3PP significantly increased extracellular ATP levels. Similarly, the nanocarriers also promoted the release of damage-associated molecular patterns (DAMPs), as evidenced by increased extracellular HMGB1 levels. Confocal laser scanning microscopy images showed a decrease in HMGB1 content within tumor cells and showed time-dependent translocation (Fig. 4h–S14a and S14c), consistent with the previous findings. Moreover, calreticulin (CRT) exposure on the cell membrane surface was increased (Fig. 4i).

Elevated levels of extracellular ATP and HMGB1 play a pivotal role in orchestrating the immune response by recruiting phagocytes and other immune effector cells to eliminate apoptotic bodies, thereby supporting an effective antitumor immune response [[35], [36], [37]]. Major histocompatibility complex class II (MHC-II)- expressing DCs present antigens to activate the adaptive immune system, making them critical components of cancer immunotherapy strategies [38]. Following treatment with drug-loaded nanoparticles (BD3PP), MHC-II expression on the surface of DCs was significantly upregulated (Fig. 4j and Fig. S14b). This finding was further validated by flow cytometry (FACS) analysis of RAW and BMDC cells (Fig. S14d and S14e), which demonstrated that BD3PP treatment significantly upregulated the expression of the M1 macrophage marker CD86, thereby indicating the activation of innate immunity. This upregulation enhanced the efficiency of antigen presentation by DCs to CD4^+^ T helper cells, thereby triggerin more robust T cell activation [39]. This process not only strengthened T cell recognition capabilities but was also fundamental for initiating a more effective anti-tumor immune response.

### 2.7 Imaging and biosafety profiles

**Fig. 5:**
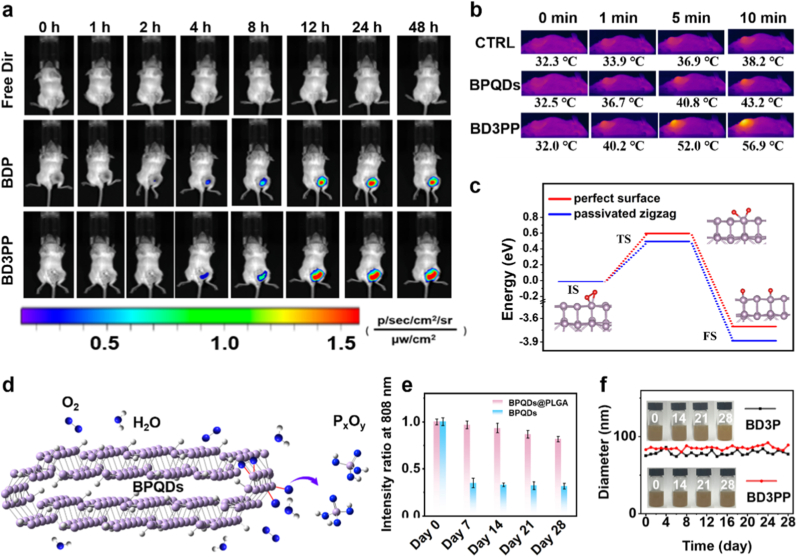
*In vivo* biodistribution of fluorescent NPs (free DIR, BDP, and BD3PP) in tumor-bearing mice and biodegradation of BPQDs. (a) Bioluminescence images of mice from different treatment groups at various time points. (b) Infrared thermal images of 4T1 tumor-bearing mice under 808 nm laser irradiation. (c) Minimum energy pathways (MEP) for O_2_ dissociation on the (010) surface (red) and at the self-passivated zigzag edge (blue) of bilayer BPQDs. Insert: snapshots of the three states; red and purple spheres represent O and P atoms, respectively. (d) Schematic illustration of BPQDs degradation. (e) Absorption intensity at 808 nm for BPQDs and BPQDs@PLGA. (f) Hydrodynamic size of NPs after storage in water. Insert: images of NPs over times.

Whole-body and *ex vivo* NIR fluorescence images were acquired using an *in vivo* imaging system (Fig. 5a and Fig. S15). As shown in Fig. 5a, free Dir exhibited minimal signal and accumulation in tumors, likely due to its inability to effectively target the tumor site *in vivo*. When **Dir** was loaded into **B**PQDs coated with **P**LGA (forming BDP), BDP progressively accumulated in both organs and tumors. Furthermore, BD3PP significantly enhanced the accumulation of Dir at the tumor site, indicating that the synthesized nanoparticles possess excellent tumor-targeting capabilities. Similar results were observed in the imaging of isolated organs presented in Fig. S16. Most of the nanomaterials were metabolized and eliminated via the liver and spleen, with the remaining fraction accumulating in tumors.

Subsequently, we evaluated the *in vivo* photothermal performance of BD3PP. As shown in Fig. 5b, the temperature at the tumor site in the BD3PP-treated group increased steadily during irradiation, reaching 56.9 °C within 10 min. In contrast, the BPQDs group exhibited a much lower temperature increase under the same conditions, which may be attributed to the enhanced tumor-targeting capability of BD3PP. These results suggest that BD3PP functions not only as an excellent photothermal agent but also as a superior tumor-targeting nanomaterial.

For the practical application of photothermal-based technologies, it is essential that the photothermal agents possess high structural stability and strong resistance to both photodegradation and chemical corrosion. However, from a biosafety perspective, it's preferable for the carrier to degrade into harmless product after accumulating at the tumor site. Previous studies have reported that the degradation of BP in water is irreversible, primarily due to the reaction of oxygen and water, which leads to the formation of oxidized phosphorus species (P_x_O_y_ anion) [40].

A comprehensive understanding of the ambient degradation of bilayer BPQDs at the atomic level was obtained by employing *ab initio* calculations within the framework of DFT. Specifically, O_2_ molecules were absorbed onto both the self-passivated zigzag edge and the (010) surface of BPQDs. O_2_ was physisorbed on BPQDs, with an adsorption energy of −1.07 eV at the self-passivated zigzag edge, which is approximately twice that of the (010) surface (−0.54 eV). This energetically favorable configuration suggests that the oxygen concentration is higher at the self-passivated zigzag edge, making this site more susceptible to degradation.

Then, the MEP for the dissociation of O_2_ on both the (010) surface and self-passivated zigzag edges of BPQDs were calculated, and the transition states were rigorously validated by frequency analysis and intrinsic reaction coordinate (IRC) calculations. After O_2_ was physisorbed onto the (010) surface of BPQDs (Fig. S17), the O-O bond length increased and P-O chemical bonds were formed, representing the initial state (IS). The chemisorbed O atom then gradually separated (transition state, TS) in an endothermic process that required overcoming an energy barrier of 0.60 eV (Fig. 5c–S18). One of the separated O atoms approached a neighboring P atom and formed two P-O bonds, resulting in the final state (FS). This was an exothermic process, releasing3.68 eV of energy. A similar process occurred at the self-passivated zigzag edge of BPQDs (Fig. S19). The energy barrier decreased to 0.50 eV, and the exothermic process released 3.86 eV of energy (Fig. S20), which is approximately 0.2 eV higher than that observed for the (010) surface. This suggests that oxidation of BPQDs at the zigzag-type edge is more energetically favorable, consistent with previous work. Subsequently, the dangling O atom on the BPQDs interacted with a H atom from H_2_O via hydrogen bonding, resulting in the detachment of the O-P from BPQDs. This process breaks the P-P bond, further degrading BPQDs into biosafe oxidized phosphorus species (Fig. 5d).

Over time, BPQDs gradually degraded within 7 days, as evidenced by a change in color in water (Fig. S21), increased zeta potentials (Fig. S22), and decreased visible light absorption (Fig. 5e and S23). In contrast, when BPQDs were encapsulated by polymers and peptides, the encapsulated BPQDs exhibited excellent stability in aqueous solution after 28 days storing in water, as indicated by unchanged hydrodynamic size (Fig. 5f), consistent color in water (Fig. 5f insert), and retained photothermal performance (Fig. S24), suggesting strong stability for further applications.

An MTT assay was conducted to evaluate the biosafety of BPQDs. As shown in Fig. S25, both newly prepared and degrade BPQDs exhibited no inhibitory effect on the proliferation of normal cell COS-7 or tumor cell 4T1. Furthermore, **3c**, BD3P, or BD3PP had minimal impact on the proliferation of normal cell COS-7 (Fig. S26) and did not induce hemolysis upon incubation with blood (Fig. S27). Taken together, these results indicate that BD3PP is stable during storage and circulation in the blood system, and is biosafe, highlighting its potential for application in tumor therapy.

### 2.8 *In vivo* antitumor effects of BD3PP in 4T1 tumor models

**Fig. 6:**
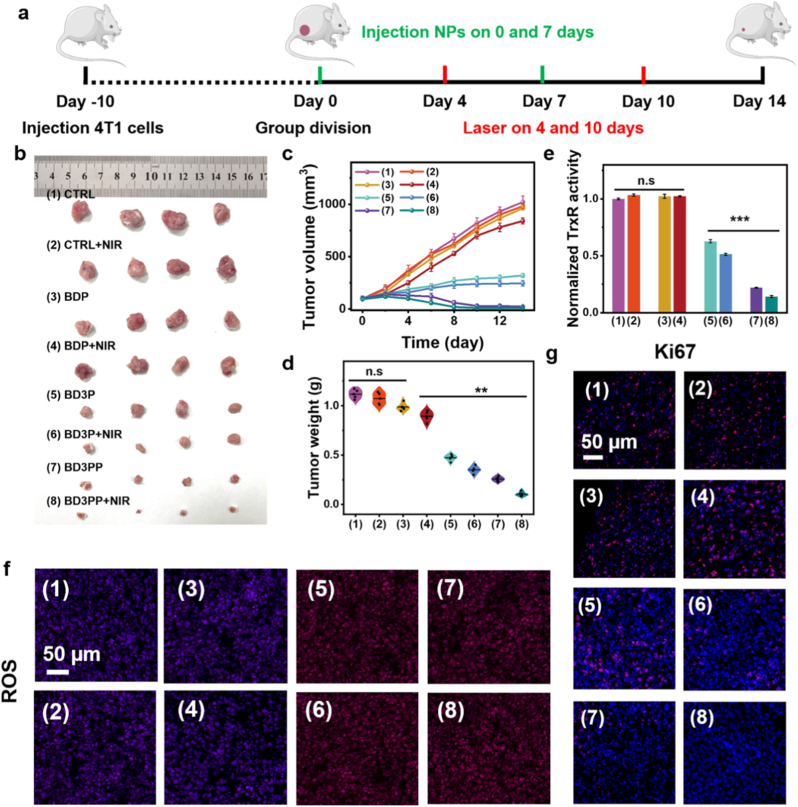
*In vivo* antitumor effects of BD3PP. (a) Schematic illustration of the treatment protocol in mice over 14 days. (b) Representative images of tumors, (c) tumor volumes, and (d) tumor weights of mice after 14 days of various treatments. (e) TrxR activity in tumor tissues following different treatments. (f) ROS staining (red) in tumor tissues from different treatment groups. (g) Ki67 staining (red) in tumor tissues from mice in different treatment groups. Nuclei: blue. (1): Control; (2): NIR; (3): BDP; (4): BDP + NIR; (5): BD3P; (6): BD3P + NIR; (7): BD3PP; (8): BD3PP + NIR.

4T1 xenograft mouse models were established to evaluate the anti-tumor efficacy of BD3PP. The treatment protocol is illustrated in Fig. 6a. Tumor-bearing mice were randomly divided into eight groups (*n* = 7) and received different treatments according to the experimental design once the tumor volume reached approximately 100 mm^3^. Tumor growth was monitored in each group throughout the treatment period (Fig. 6b–d). Each component of BD3PP acted synergistically to inhibit tumor growth. Among the treatments without laser irradiation, BD3PP demonstrated the most pronounced anti-tumor effect. When combined with NIR light irradiation, the therapeutic effect was further enhanced through the synergistic effects of PTT and PDT, thereby achieving highly efficient and precise tumor treatment.

Owing to the higher level of arsenic accumulation in tumors (Fig. S28) and the photoimmunotherapeutic effects of BPQDs, the BD3P and BD3PP cohorts exhibited a significant decrease in TrxR activity (Fig. 6e) and a marked increase in ROS levels (Fig. 6f), which is consistent with the cellular results (Fig. 3).

To further validate its superior anti-tumor efficacy, immunofluorescence staining for the proliferation marker Ki67 was performed. Compared to BDP and BD3P, BD3PP treatment significantly reduced the number of Ki67-positive cells within tumors (as indicated by red fluorescence), indicating a more potent inhibitory effect on tumor cell proliferation (Fig. 6g). These results strongly suggest that the synergistic combination of targeted therapy, PTT, and PDT effectively inhibited tumor progression and, in some cases, even resulted in complete tumor elimination.

This biosafety of BD3PP was subsequently evaluated. Continuous monitoring of the body weight throughout the treatment period showed no significant changes (Fig. S29). Comprehensive blood, liver, and kidney function tests were using blood samples. Hematological parameters (Fig. S30) and liver and kidney function indices (Fig. S31) remained within normal ranges following BD3PP treatment, indicating the absence of significant infection or inflammation. Major organs, including the heart, liver, spleen, lungs, and kidneys, were collected for further analysis. H&E staining of these organs revealed no evidence of necrosis or swelling (Fig. S32), further demonstrating the excellent biocompatibility of BD3PP *in vivo*. Due to the high efficacy and favorable biosafety profile of BD3PP, the survival rate of treated mice was maintained for over 50 days (Fig. S33).

### 2.9 BD3PP-induced antitumor immunity *in vivo*

**Fig. 7:**
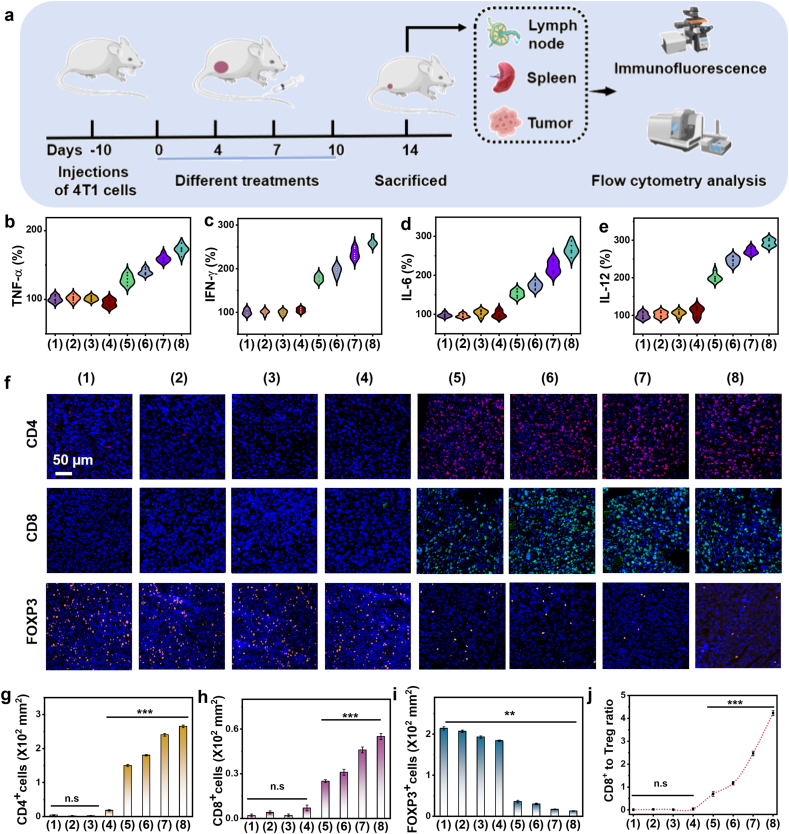
**(**a) Schematic illustration of tumor inoculation and treatment timeline. (b–e) Cytokine levels of TNF-α, IFN-γ, IL-6, and IL-12 in serum samples collected from mice isolated from different groups on day 14. (f–g) Representative immunofluorescence staining images and corresponding quantitative analysis of CD4^+^ cells (red), CD8^+^ cells (green), and FOXP3^+^ cells (orange) in tumor tissues.

Furthermore, typical cytokines, including TNF-α, IFN-γ, IL-6, and IL-12, were found to be upregulated (Fig. 7a–e) following BD3P or BD3PP treatment, with/without laser irradiation. Laser irradiation further increased the expression of these cytokines, thereby significantly enhancing the immune response. In contrast, BPQDs treatment at this dosage had minimal effect on immune regulation.

Furthermore, CD4^+^ and CD8^+^ T cells, as well as FOXP3^+^ regulatory T cells, in tumor tissues were stained to assess immune changes following these treatments (Fig. 7f–j). Immunofluorescence images revealed a marked increase in the fluorescence signals of both CD4^+^ and CD8^+^ cells, suggesting effective activation of anti-tumor immunity. Concurrently, a decrease in the fluorescence intensity of FOXP3^+^ cells was observed. Given that FOXP3 is a principal marker of immunosuppressive regulatory T cells, its decreased expression implied a reduction in immunosuppression within the tumor microenvironment, thereby promoting enhanced immune recognition and attack against tumor cells [41].

In this study, a multifunctional and traceable nanoparticle (BD3PP) was synthesized by integrating a self-passivated bilayer BPQD core, PD-L1 antibody conjugation, TrxR inhibitor (3c) loading, and a stimulus-responsive fluorophore (Dir). The composition, structure, and biomedical properties of BD3PP were systematically investigated using DFT and TD-DFT calculations, molecular docking analyses, as well as comprehensive *in vitro* and *in vivo* experiments. BD3PP integrates targeted therapy, PTT, PDT, and immunotherapy, as well as photothermal and NIR fluorescence imaging capabilities.

BD3PP exploits the specific targeting of PD-L1 on tumor cells via the PD-L1 antibody ^D^PPA-1. Upon light radiation, BPQDs efficiently converted optical energy into heat, enabling PTT with a photothermal conversion efficiency of 25.2 %. The generated heat signal supported effective *in vivo* photothermal imaging, enabling visualization of the therapeutic process and tumor localization. TD-DFT calculations revealed that the synthesized ∼2.3 nm bilayer BPQDs possessed self-passivated edges. Compared to non-self-passivated BPQDs, the self-passivated BPQDs transferred optical energy to O_2_ to generate ^1^O_2_ much more efficiently, with the SOC value exceeding 20 cm^−1^, which is 10-fold higher than non-self-passivated BPQDs. Because of the presence of 4 dark states, electrons can first transition to these states, thereby increasing the possibility of intersystem crossing and supporting ^1^O_2_ generation for strong type II PDT. The self-passivated edges enormously increase the transferring of optical energy, promoting the wide usage in optical materials.

Molecular docking and mass spectrum analyses demonstrated that molecule **3c** was covalently bound to TrxR, one of the most important redox enzymes. The covalent binding has higher selectivity than non-covalent binding, supporting the highly selectivity of our nanomaterials and mere toxic side effects. As a result, **3c** exclusively inhibited TrxR activity and induced oxidative stress, leading to elevated levels of ROS, including **·** $O_{2}^{‐}$ and H_2_O_2_. In synergy with PTT and PDT effects of BPQDs, **3c** induced tumor cell apoptosis and ICD, thereby promoting the polarization of TAMs towards the M1 phenotype. In combination with immunosuppression relief mediated by ^D^PPA-1, BD3PP significantly enhanced anti-tumor immunity by remodeling the TME.

DFT calculations demonstrated O_2_ is preferentially adsorbed at the self-passivated zigzag edge of BPQDs rather than on the (010) surface. The adsorbed O_2_ gradually dissociated to form P-O bonds, which subsequently interacted with H_2_O to generate phosphoric acid, resulting in the biosafe degradation of BPQDs. Encapsulation within PLGA effectively protected BPQDs from exposure to O_2_ and H_2_O, thereby preserving their stability during storage and systemic circulation prior to reaching tumor sites. This biodegradable nanomaterial provides an outstanding biosafe candidate for more bio-application.

Experimental results in 4T1 tumor-bearing mice demonstrated that two administrations of BD3PP achieved over 90% tumor inhibition rate by the synergistic combination of multiple therapeutic modalities. Notably, ICD contributed to systemic antitumor immune activation, thereby amplifying the overall therapeutic efficacy. Overall, this work provides a promising strategy for the highly efficient treatment of tumors using multifunctional, biosafe nanodrugs.

## 3 Conclusion

In this study, multifunctional BD3PP were constructed by PLGA encapsulating BPQDs, thioredoxin reductase inhibitor **3c,** the fluorescent dye Dir, and conjugated PDL1 antagonist for synergistic therapy and imaging. PDL1 antagonist guided BD3PP to tumor cell, enabling controlled release of the 3 therapeutic agents into tumor cells. Near-infrared light energy was transferred by self-passivated bilayer BPQDs into heat for photothermal and type II photodynamic therapy. Targeted inhibition of thioredoxin reductase by **3c** induced oxidative stress, which synergistically with PDT therapy induced immunogenic cell death (ICD) to reprogram the tumor microenvironment to clear tumors. The near-infrared fluorescent dye Dir and photothermal effect by BPQDs supported real-time imaging *in vitro* and *in vivo*. The innovative multifunctional and biosafe nanomaterial exhibits remarkable potential for cancer theranostic.

Compared with recently reported BP-based or immune-modulating nanoplatforms, BD3PP is distinguished by the integration of passivated BPQDs with enhanced photothermal/photodynamic performance, targeted immune checkpoint blockade, and redox-based chemotherapy within a single platform. Rather than relying solely on the intrinsic photothermal or photodynamic effects of BP, BD3PP achieves synergistic tumor eradication through coordinated photothermal, photodynamic, and ICD-mediated immunotherapeutic mechanisms, thereby offering improved functional integration and therapeutic potential.

## 4 Materials and methods

### 4.1 Materials

All reagents were of analytical grade and purchased from Aladdin (Shanghai, China) unless otherwise mentioned. BP crystals were purchased from the Smart-Elements (Vienna, Austria). 1-Methyl-2-pyrrolidinone (NMP, 99.5%, anhydrous), LLC (Santa Barbara, USA). Polylacticcoglycollic acid (PLGA, 50:50, MW: 40,000–70,000), polyvinyl alcohol (PVA, MW: 9000–10,000) and DCM were purchased from the Sigma-Aldrich (Santa Barbara, USA). Dir fluorescence dye was purchased from the Innova Biosciences (UK). PLGA12000-PEG3400-maleimide (LA:GA = 50:50) was purchased from Huateng Pharma (Hunan, China). Cysteine-terminated PD-L1 antagonist peptide (NYSKPTDRQYHF) was synthesized by GL Biochem Co., Ltd. (Shanghai, China). 5,5-Dimethyl-1-pyrroline N-oxide (DMPO), 2,2,6,6-Tetramethylpiperidine (TEMP), 1,3-diphenylisobenzofuran (DPBF), 2,7 -dichlorofluorescein diacetate (DCFH-DA), methyl thiazolyl tetrazolium (MTT) and Tris(2-carboxyethyl) phosphine (TCEP) were bought from Aladdin Bio-Chem Technology Co., Ltd. (Shanghai, China). Cyt-*c* ELISA Kit was bought from Jiyinmei bio. (Wuhan, China). All the chemicals used in this study were at the analytical reagent grade.

### 4.2 Synthesis of BPQDs

BPQDs (**b**lack **p**hosphorus **q**uantum **d**ots) were prepared using a simple liquid exfoliation technique as described in the literature. In brief, 20 mg of bulk BP powder was dispersed in 20 mL of NMP (1-Methyl-2-pyrrolidinone) and sonicated with a probe sonicator (ultrasonic frequency: 19–25 kHz) for 4 h (period of 2 s with the interval of 4 s) at a power of 1200 W. The resulting mixture was further sonicated in an ice bath for 12 h at a power of 300 W. The dispersion was then centrifuged at 7000 rpm for 20 min, and the supernatant containing the BPQDs was collected.

### 4.3 Preparation of BP/Dir/3c@PLGA NSs (BD3P)

BPQDs/PLGA nanospheres (NSs) were prepared using an oil-in-water emulsion solvent evaporation method. Briefly, the previously prepared BPQDs (1 mg) were redispersed in PLGA-PEG-MAL DMSO solution (10 mg/mL, 1 mL) containing 1 mg 3c and 1 mg Dir. After sonication for 5 min by an ultrasonic homogenizer, the mixture was re-suspended in 0.5% (*w*/*v*) PVA aqueous solution (10 mL) and sonicated again for 5 min. The emulsion was further stirred overnight at the room temperature to remove the residual DMSO. The resulting nanospheres were collected by centrifugation at 7000 rpm for 15 min and washed twice with deionized water.

### 4.4 Preparation of BP/Dir/3c@PLGA NSs -^D^PPA-1(BD3PP)

PLGA-PEG-MAL nanoparticles loaded with BP/Dir/3c were prepared using the emulsion/solvent evaporation method. For peptide surface conjugation, an appropriate amount of tris (2-carboxyethyl) phosphine (TCEP) and cysteine-terminated PD-L1 antagonist peptide were added to the nanoparticle solution (1 mg/mL) and stirred at 4 °C for 12 h [42]. The resulting mixture was washed and concentrated using ultracentrifuge filters (2 kDa MWCO) at 4 °C, and then redispersed in PBS to obtain nanoparticles BP/Dir/3c@PLGA NSs -^D^PPA-1(BD3PP, the last P was the abbreviation of peptide).

### 4.5 Characterizations of BD3PP

TEM and high-resolution TEM (HRTEM) images of the nanoparticles were acquired by a JEM-F200 transmission electron microscope (JEOL, Japan). AFM imaging was performed on a Dimension Icon AFM (Bruker, Germany). The hydrodynamic diameter and ζ-potential were measured by a Zetasizer Nano ZS90 (Malvern, UK).

### 4.6 Photothermal performance

The detailed calculation of photothermal conversion efficiency was performed as described in a previous study [43]. Briefly, the concentration of BD3PP was 1.0 mg mL^−1^ and the power density of the 808 nm laser was 1.0 W cm^−2^. An infrared thermal imager was used to record the temperature change of the solution under laser irradiation. For both *in vitro* and *in vivo* experiments, laser irradiation was carried out at a wavelength of 808 nm with a power density of 1.0 W cm^−2^ for 10 min.

### 4.7 Detection of ROS

DPBF was employed as a photodegradation probe to evaluate the *in vitro* photodynamic effect of BPQDs, and the light absorption of samples was recorded by UV–vis spectroscopy. To further explore the photodynamic activity of BPQDs, electron spin resonance (ESR) spectra was performed using TEMP as the ^1^O_2_ trapping agent. Intracellular total ROS were detected using the fluorescent probe DCFH-DA. For the intracellular ^1^O_2_ detection, the SOSG probe was employed, and cellular images were acquired by CLSM. Additionally, intracellular H_2_O_2_ generation capability was assessed using an assay kit.

### 4.8 Calculation details

All calculations were performed using the ORCA software package [44,45] at the DFT level of theory. Ground-state structures were optimized without any constraints using the B3LYP hybrid exchange-correlation functional [46]. The D3 method with Becke–Johnson (BJ) damping [47] was employed to account for van der Waals interactions, which are crucial for accurately describing non-covalent interactions in this system. The standard Pople 6-31G∗ basis set was used for all atoms. The ωB97M − V functional and def2-TZVP basis set were used for all energy calculations. Electronic excitations and SOC were calculated from the optimized ground-state structures using TD-DFT. In TD-DFT calculations, the B3LYP functional and def-TZVP basis set [48] were employed. Post-processing and analysis of the quantum chemical calculations were performed using Multiwfn [49,50].

### 4.9 Molecular docking

Molecular docking was performed to investigate the interaction between 3c and TrxR (Ledock, http://lephar.com). The crystal structure of TrxR (PDB:3EAN) was obtained from the Protein Data Bank (http://www.rcsb.org/pdb/home/home.do). The molecular docking procedure was carried out 50 times randomly, and all other parameters were default values. Docking images with molecular interactions were generated by Pymol software (http://www.pymol.org).

### 4.10 PD-L1 blocking assay

4T1 cells were seeded in six-well culture plates and cultured for 24 h. The cells were then incubated with PBS, free ^D^PPA-1, or BD3PP for 30 min. Later, the cells were stained with α-PD-L1-APC, and the binding of α-PD-L1-APC to cells was measured by an Accuri™ C6 Plus flow cytometer (Becton, Dickinson and Company, USA).

### 4.11 Drug loading content (LC) and encapsulation efficiency (EE)

After synthesis, BD3PP samples were dried and accurately weighed to obtain the total mass (m). The arsenic (As) content in the samples was subsequently determined using HPLC (High Performance Liquid Chromatography). The encapsulation efficiency (EE%) and drug loading content (LC%) were calculated according to the formulas provided in Table 1.

### 4.12 Western blotting analysis

After treatment, 4T1 cells were collected and lysed in RIPA buffer. Protein concentration was examined using a BCA Protein Assay Kit. Equal amounts of total protein (30 μg) for each group were subjected to sodium dodecyl sulfate-polyacrylamide gel electrophoresis (SDS-PAGE), followed by Western blotting. Primary antibodies (Abs) were used as indicated, with GAPDH serving as the loading control. All antibodies were used at a 1:1000 dilution.

### 4.13 Detection of ICD

4T1 cells were cultured in six-well plates at a density of 6 × 10^6^ cells/well for 12 h. Then fresh medium containing nanomaterials was added at a concentration of 50 μg mL^−1^ for 4 h, followed by irradiation by a laser (808 nm, 1.0 W cm^−2^, 10 min) or no irradiation. For the control group, an equal volume of fresh medium without nanomaterials was added. After 24 h of incubation, the cell culture medium was collected, and extracellular HMGB1 levels were measured by an HMGB1 ELISA kit according to the manufacturer's instructions. ATP secretion was measured by an ATP detection kit. Furthermore, cells were incubated overnight with primary antibodies against CRT and HMGB1 according to the manufacturer's protocol. Then, the cells were incubated with corresponding secondary antibodies (1 h, 25 °C), and nuclei were stained with DAPI. Fluorescence images were acquired using a Nikon Eclipse C1 fluorescence microscope and a Nikon DS-U3 imaging system (Japan).

### 4.14 Hemolysis assay

0.5 mL of the sample was mixed with an equal volume of 2 % erythrocyte suspension and incubated in a shaker at 37 °C for 4 h, followed by centrifugation for 15 min (4 °C, 3000 rpm). PBS and ddH_2_O were employed as the negative and positive controls, respectively. The absorbance at 540 nm was then measured for 200 μL of the supernatant. The hemolysis rate (%) was determined using the following equation:

Hemolysis (%) = $\frac{\text{Abs}\left(\text{Sample}\right)-\text{Abs}\left(\text{PBS}\right)}{\text{Abs}\left(\text{dd}H_{2}O\right)-\text{Abs}\left(\text{PBS}\right)}$ × 100%

### 4.15 Cell culture

4T1 and COS-7 cells lines were obtained from the China Center for Type Culture Collection (CCTCC). Both cell lines were cultured in RPMI-1640 supplemented with 10% fetal bovine serum and 1% penicillin/streptomycin, and maintained in a humidified incubator at 37 °C with 5% CO_2_.

### 4.16 Experimental mouse models

Four-week-old BALB/c mice (female) were purchased from Beijing HFK BIOSCIENCE. The mice were housed under standard conditions. 4T1 cells (2 × 10^6^/cells) was subcutaneously injected into the right hind leg of mice. Mice were randomly divided into groups when the tumors reached ∼100 mm^3^. On day 0, the mice were randomly assigned to eight treatment groups: (1) PBS, (2) PBS + NIR, (3) BDP, (4) BDP + NIR, (5) BD3P, (6) BD3P + NIR, (7) BD3PP, (8) BD3PP + NIR. The mice were intravenously injected with different formulations (1 mg kg^−1^ of nanoparticles) on day 0 and day 7. Meanwhile, the tumors of mice in groups (2), (4), (6), and (8) were irradiated with an 808 nm laser (1.0 W cm^−2^, 10 min) on day 4 and day 10. Tumor growth (*V* = 0.5 × Length × Width^2^) and body weight of mice were monitored every two days. Mice were sacrificed on day 14, and fresh tumors were harvested for Ki67, ROS, ATP, and HMGB1 assay. Major organs were collected for H&E staining. Blood samples were collected for routine hematological analysis. Serum was collected for analysis of liver and kidney function indices, as well as for the detection of IL-6 and IL-12 using ELISA kits according to the manufacturer's instructions.

### 4.17 Quantification and statistical analysis

All experiments were performed in at least three replicates. The data were presented as mean ± SD. Statistical significance was analyzed using the one-way ANOVA, and significance was indicated by asterisks in all Figures (∗∗∗*P* < 0.001, ∗∗*P* < 0.01, ∗*P* < 0.05, ns, not significant).

## Ethics approval and consent to participate

All animal studies were conducted in accordance with the standards outlined in China's National Regulations for the Care and Utilization of Laboratory Animals and after approval by the ethical committee of Tiangong University.

## CRediT authorship contribution statement

**Tingting Liu:** Conceptualization, Investigation, Methodology, Writing – original draft. **Wenyan She:** Investigation, Methodology, Writing – review & editing. **Ruili Du:** Investigation, Methodology, Writing – review & editing. **Yali Bao:** Investigation, Methodology, Writing – review & editing. **Zhibin Guo:** Data curation, Formal analysis, Software, Validation. **Qichao Gao:** Data curation, Formal analysis, Software, Validation. **Hanping Li:** Data curation, Formal analysis, Software, Validation. **Pengfei Suo:** Formal analysis, Methodology, Writing – review & editing. **Yi Liu:** Funding acquisition, Project administration. **Yujiao Liu:** Conceptualization, Funding acquisition, Methodology, Project administration, Writing – review & editing.

## Declaration of competing interest

The authors declare that they have no known competing financial interests or personal relationships that could have appeared to influence the work reported in this paper.

## Data Availability

No data was used for the research described in the article.

## Abbreviations

***BPQDs***: black phosphorus quantum dots
***ICB***: immune checkpoint blockade
***TME***: tumor microenvironment
***TNBC***: triple-negative breast cancer
***TAMs***: tumor-associated macrophages
***ICD***: immunogenic death
***BP***: black phosphorus
***PTT***: photothermal therapy
***DFT***: Density functional theory
***TD-DFT***: time-dependent density functional theory
***HOMO***: the highest occupied molecular orbital
***LUMO***: the lowest unoccupied molecular orbital
***SOC***: spin-orbit coupling
***TrxR***: thioredoxin reductase
***ROS***: Reactive oxygen species
***•OH***: Hydroxyl radicals
***NIR***: Near-infrared
