# Supplementary material for: Self-passivated bilayer black phosphorus QDs based multifunctional nanoparticles for tumor immune reprogramming
Source: Mater Today Bio. 2026 Feb 2;37:102862. doi: 10.1016/j.mtbio.2026.102862 (PMC12914194; doi:10.1016/j.mtbio.2026.102862)
Supplement: Multimedia component 1 [file mmc1.docx]

Supporting Information


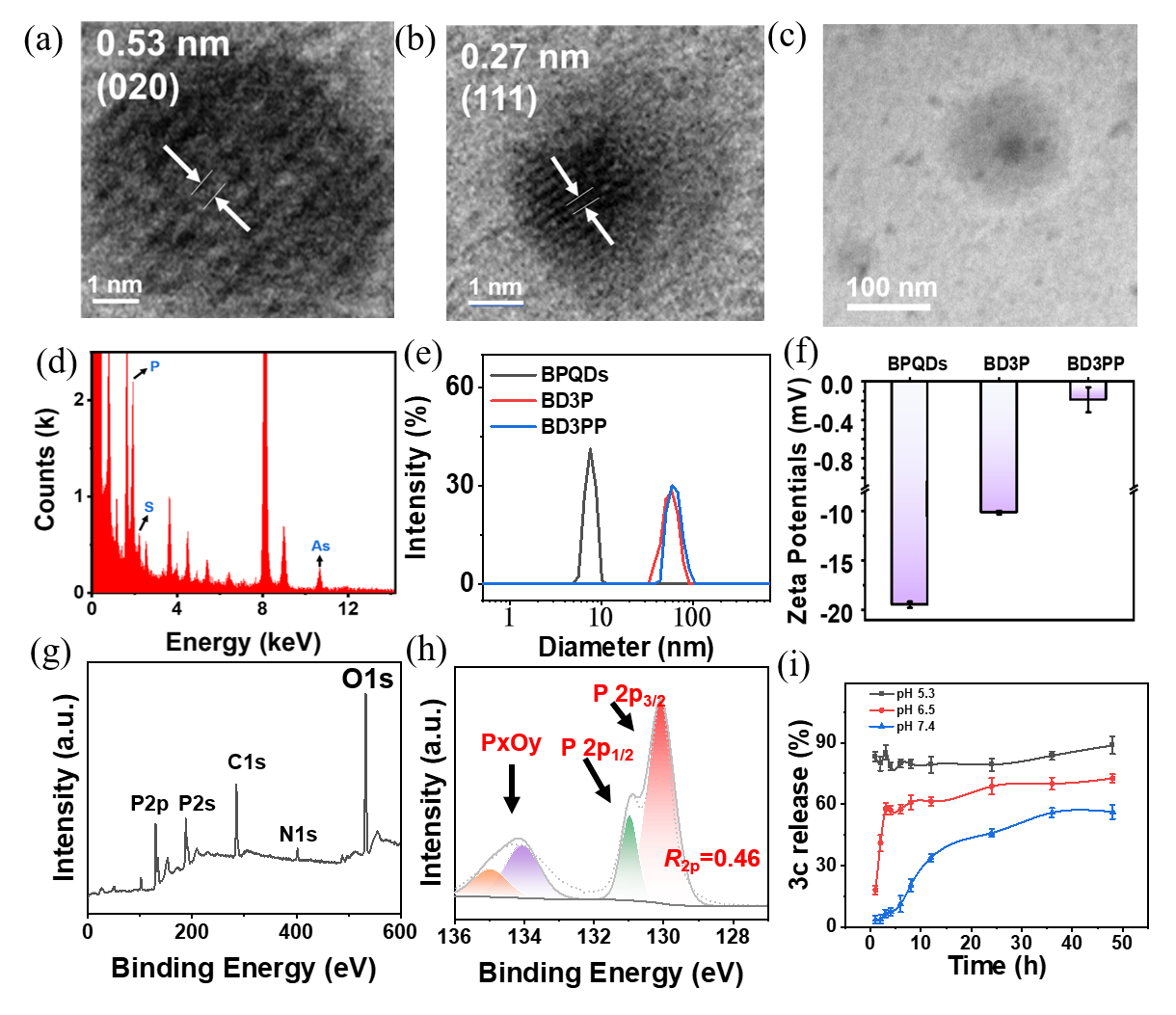


**Figure S1.** (a, b). High-resolution TEM image of BPQDs. (c) TEM images of BD3PP. (d) Hydrodynamic diameter of BPQDs, BD3P, and BD3PP. (e) Elemental analysis of BD3PP. (f) ζ-potential of BPQDs, BD3P, and BD3PP. (g, h) XPS spectra of passivated BP quantum dots (BPQDs) dispersed in aqueous solution. (i) The 3c controlled release of BD3PP triggered by different pH levels.


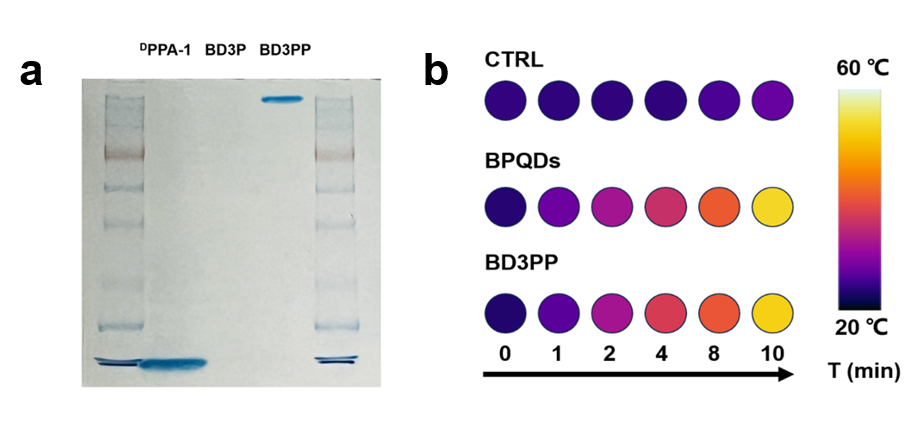


**Figure S2.** (a) SDS-PAGE protein gel stained by Coomassie Brilliant Blue. (b) Thermal imaging of water, BPQDs, and BD3PP under 808 nm laser irradiation (density: 1.0 W cm^-2^).


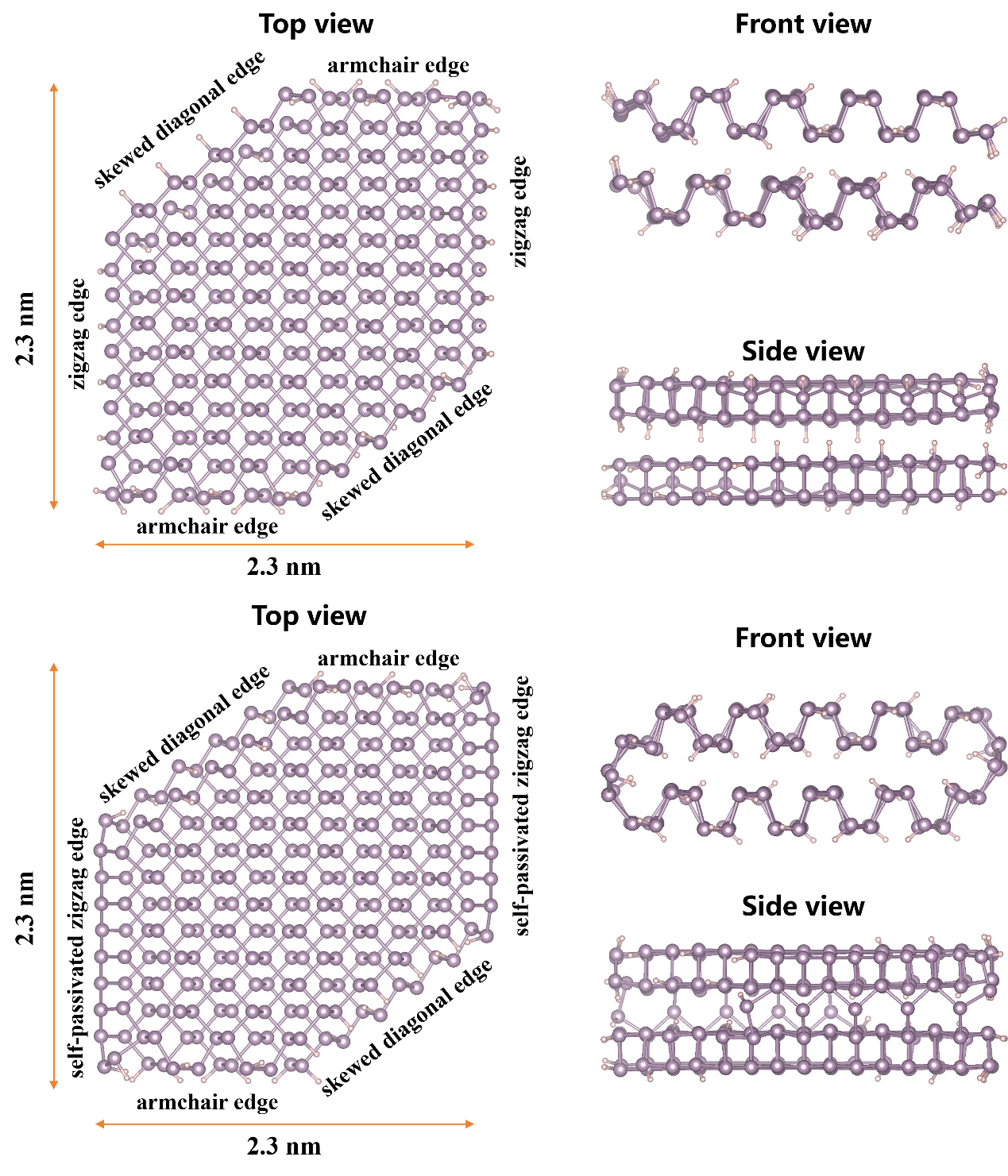


**Figure S3.** Views of non-self-passivated BPQDs (top) and self-passivated BPQDs (bottom).


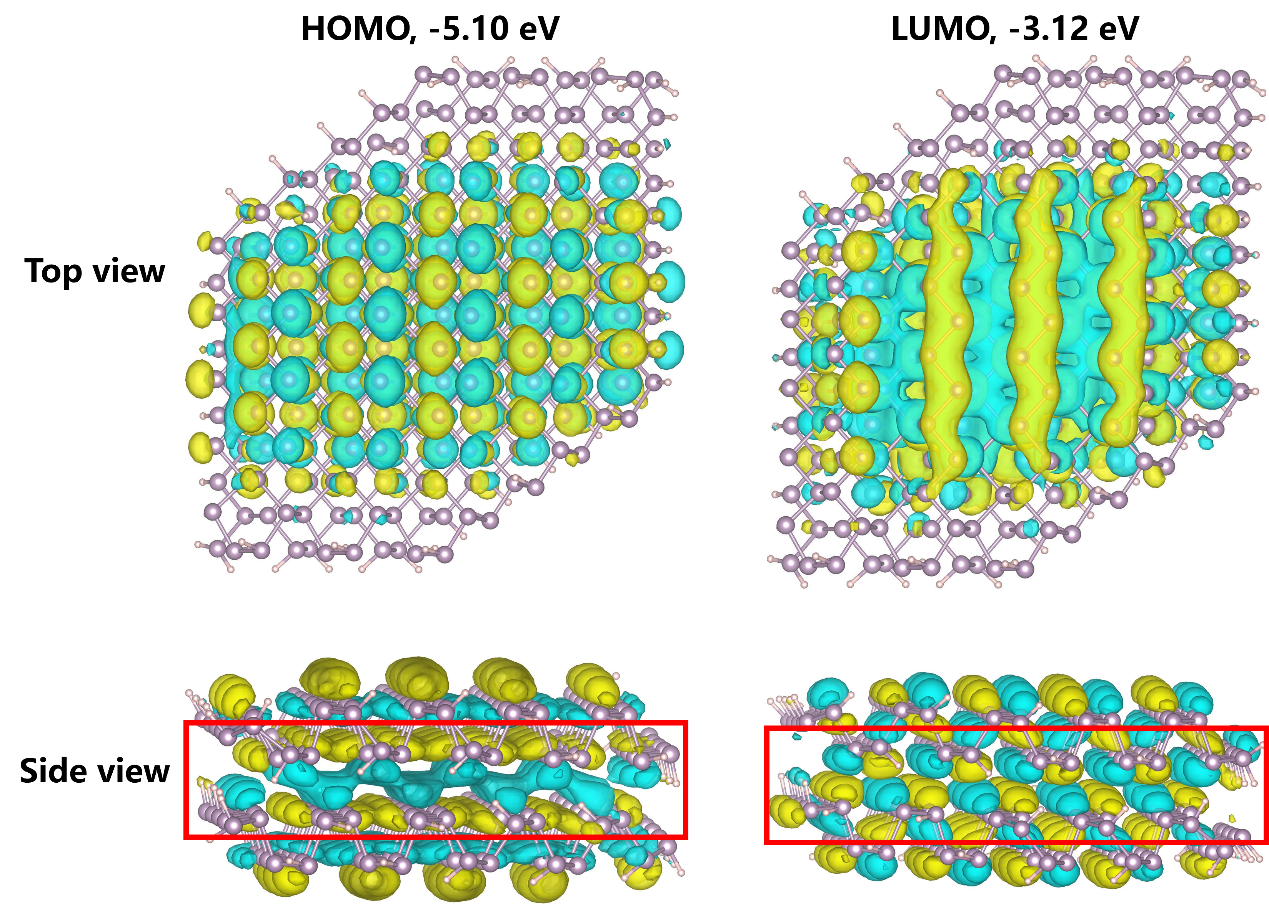


**Figure S4.** Molecular orbitals and energy levels of model 1. In real space, the HOMO and LUMO states were nearly extended throughout the bilayer (top). A clear anti-bonding feature was visible in the interlayer region for the HOMO (bottom left). A bonding feature was also found in the LUMO (bottom right), where it observed not between the layers but across the troughs.


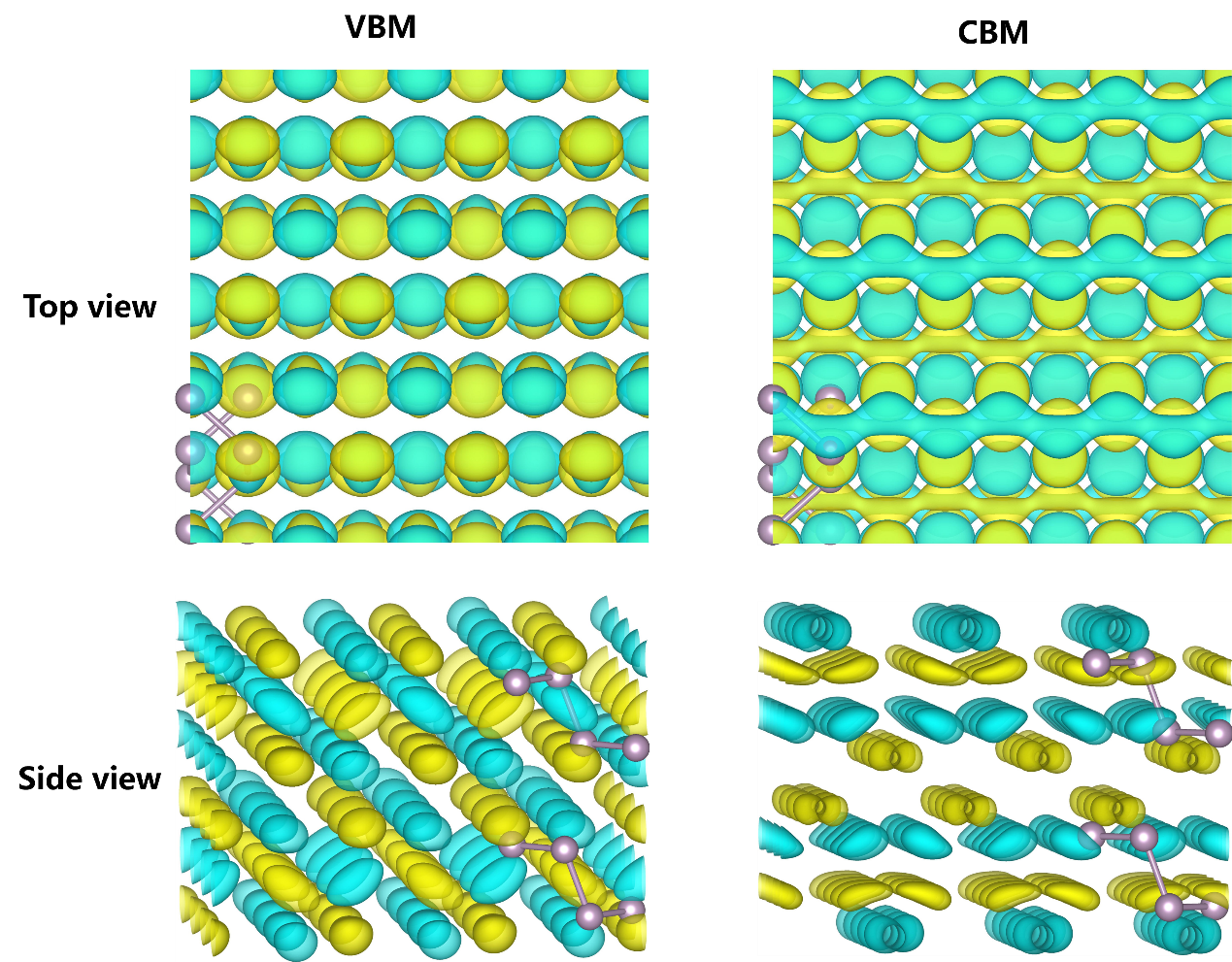


**Figure S5.** Wavefunction of the Γ point of black phosphorus.


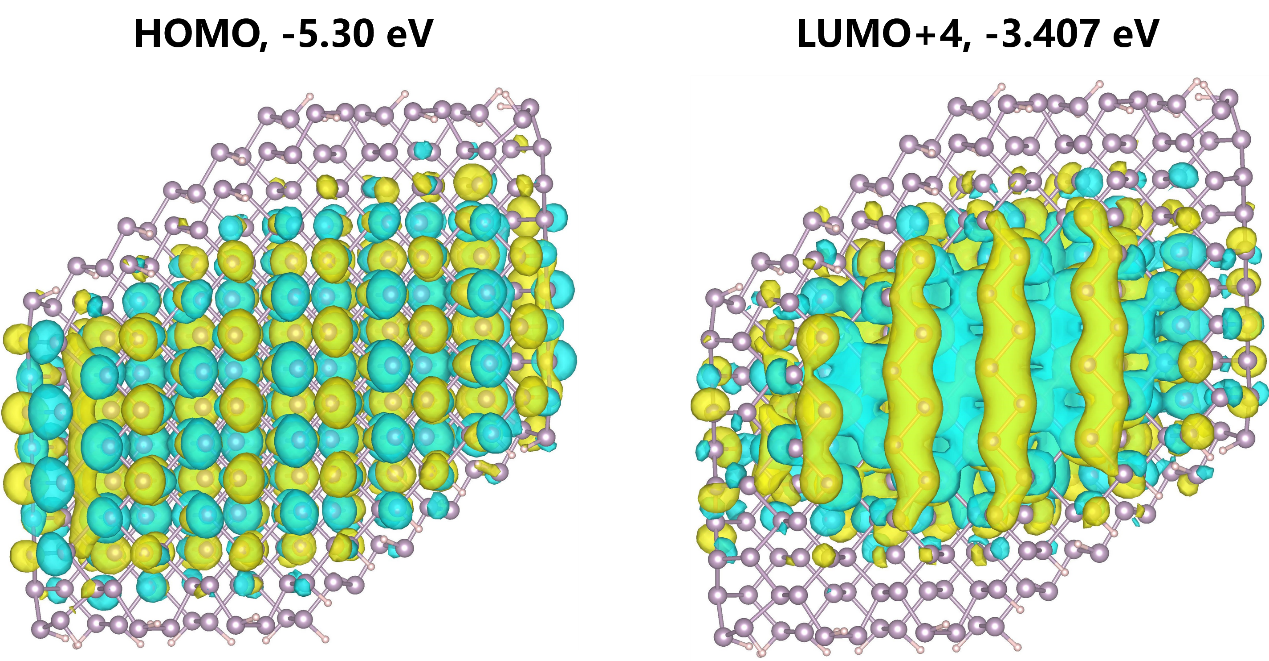


**Figure S6.** Molecular orbitals and energy levels of model 2.


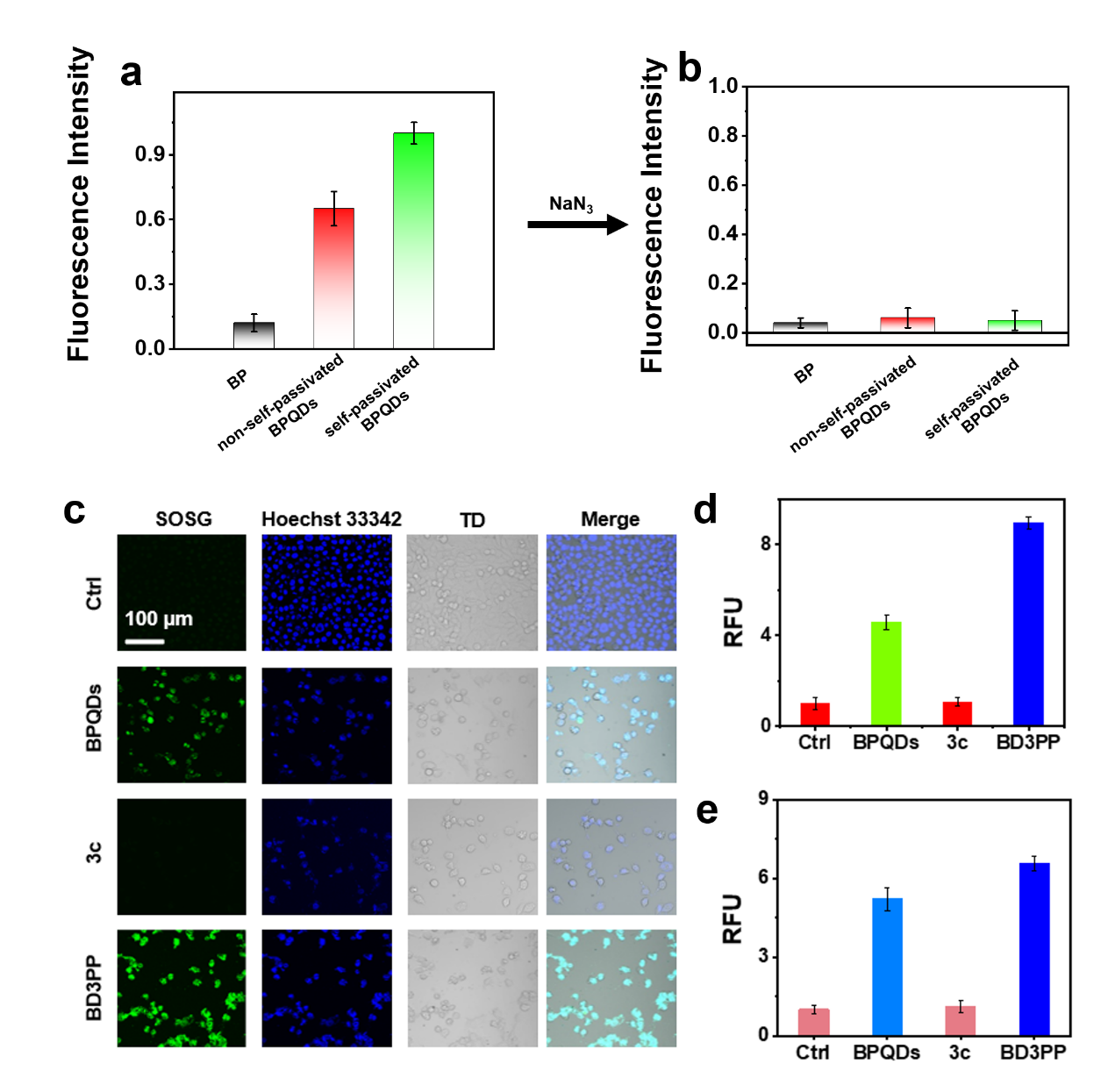


**Figure S7.** (a, b) The Fluorescence intensity of DCFH-DA. (c) Confocal images of 4T1 cells treated with BPQDs, 3c, and BD3PP, probed by SOSG. Green: SOSG for ^1^O_2_; Blue: Hoechst 33342 for cell nucleus. (d) Relative fluorescent intensity of ^1^O_2_ detected by CLSM. (e) Relative fluorescence intensity of ^1^O_2_ detected by fluorescence spectrophotometry.


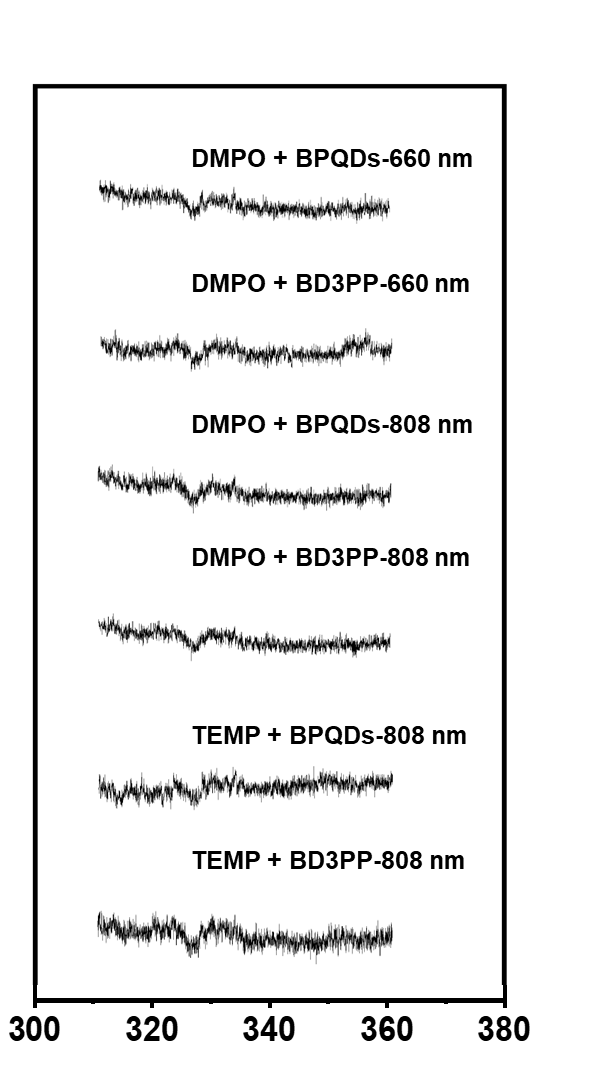


**Figure S8.** ESR spectra of BPQDs and BD3PP after 5 min of 660 nm or 808 nm laser irradiation, using DMPO as the scavenger for **·**$\text{O}_{\text{2}}^{\text{-}}$ and **·**OH, or TEMP as the ^1^O_2_ probe.


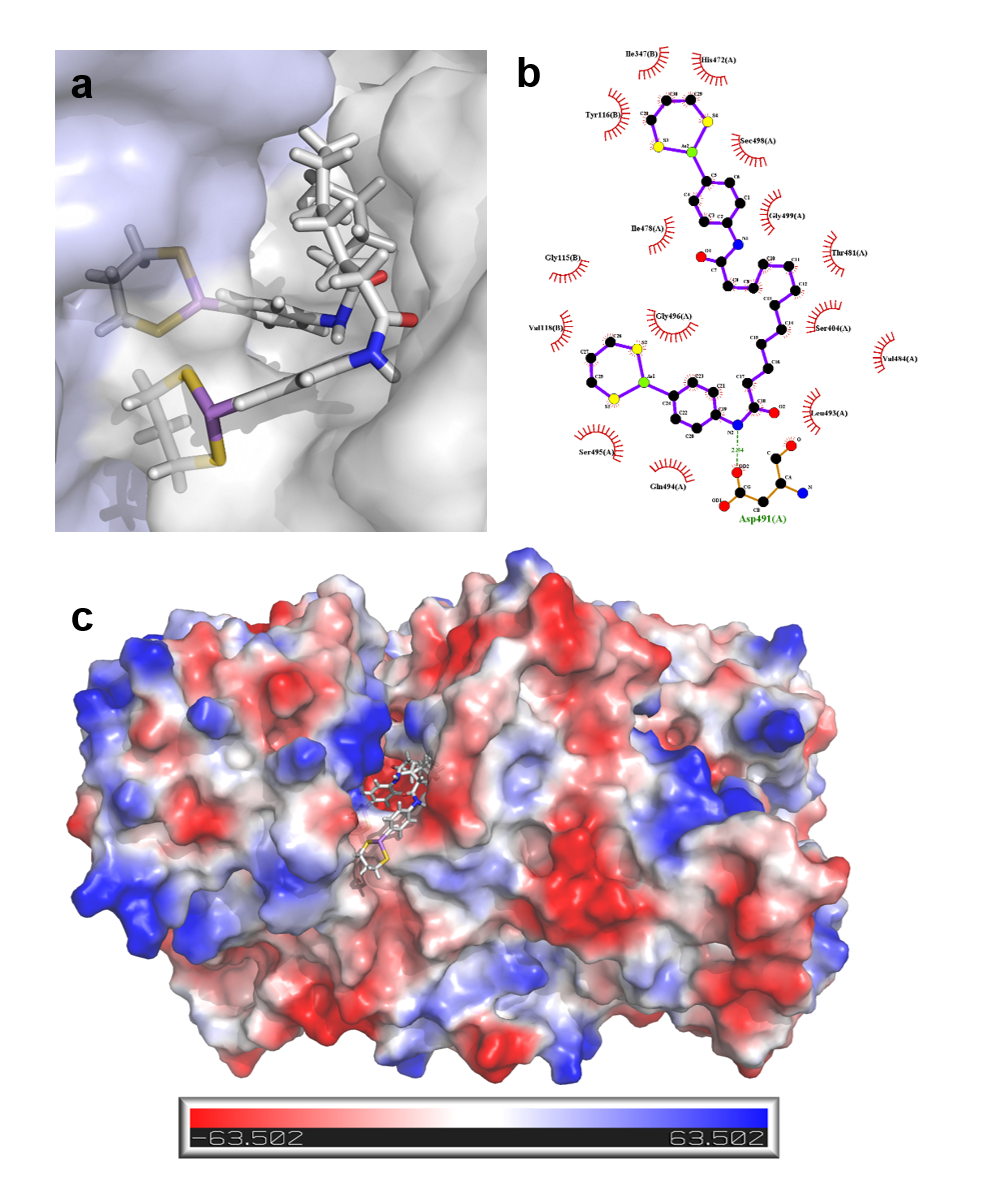


**Figure S9.** Molecular docking results of TrxR protein with 3c.


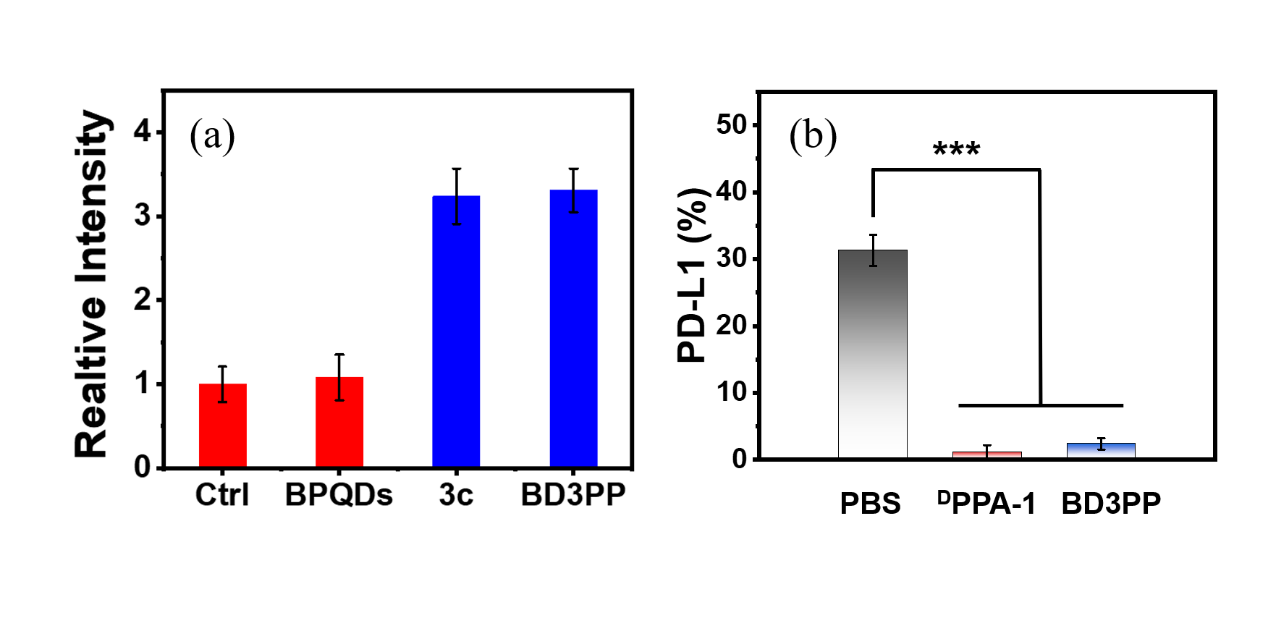


**Figure S10.** (a) H_2_O_2_ content in 4T1 cells after incubation with BPQDs, 3c, and BD3PP. (b) Flow cytometric analysis of the PD-L1 blockade effect of PBS, free DPPA-1, and BD3PP on 4T1 cells.


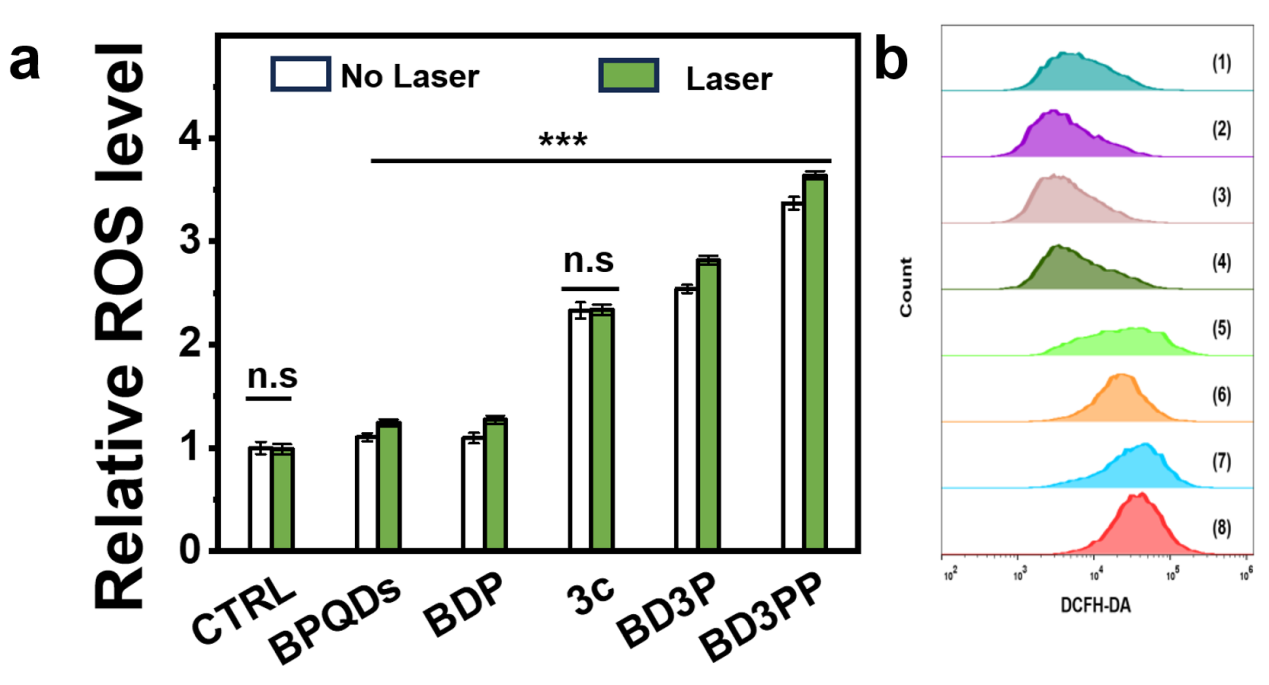


**Figure S11.** (a) Relative ROS levels in 4T1 cells of figure 5A. (b) FCAS analysis of ROS in 4T1 cells under different treatments (ROS probe: DCFH-DA). (1): Control; (2): NIR; (3): BDP; (4): BDP+NIR; (5): BD3P; (6): BD3P+NIR; (7): BD3PP; (8): BD3PP+NIR.


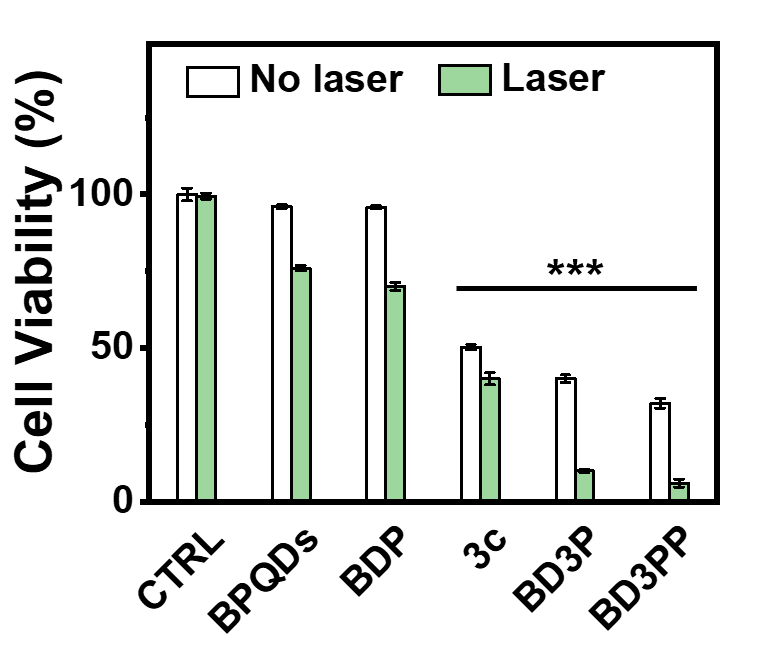


**Figure S12.** Cell viability under different treatments.


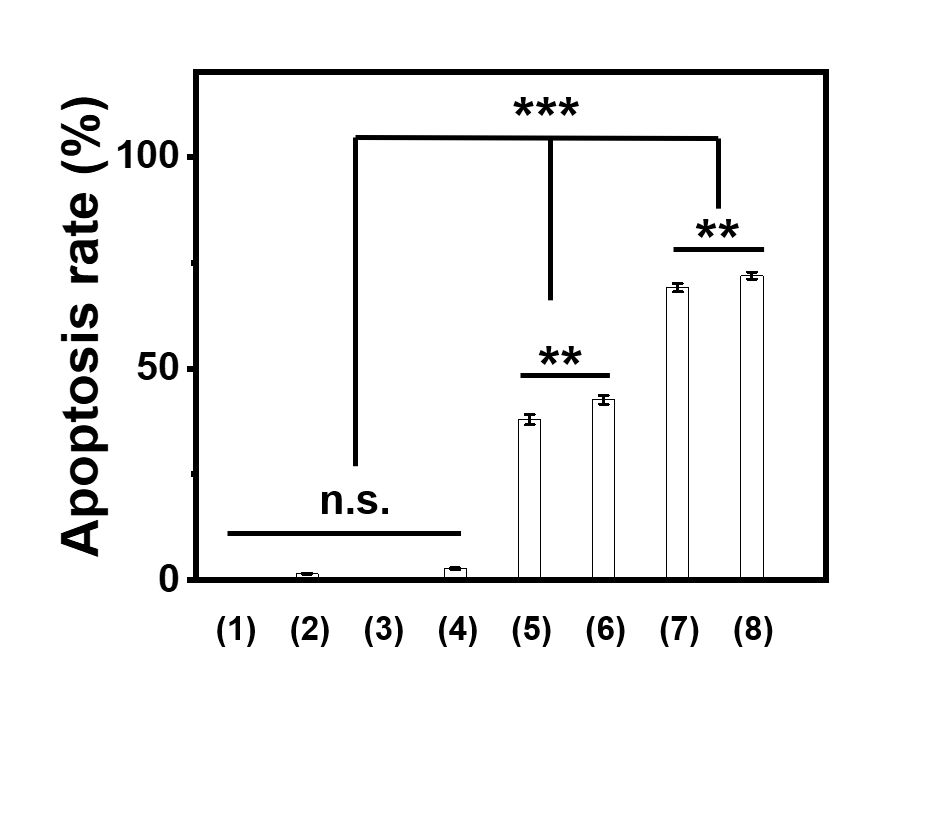


**Figure S13.** Relative percentages in 4T1 cell groups of Figure 4E.


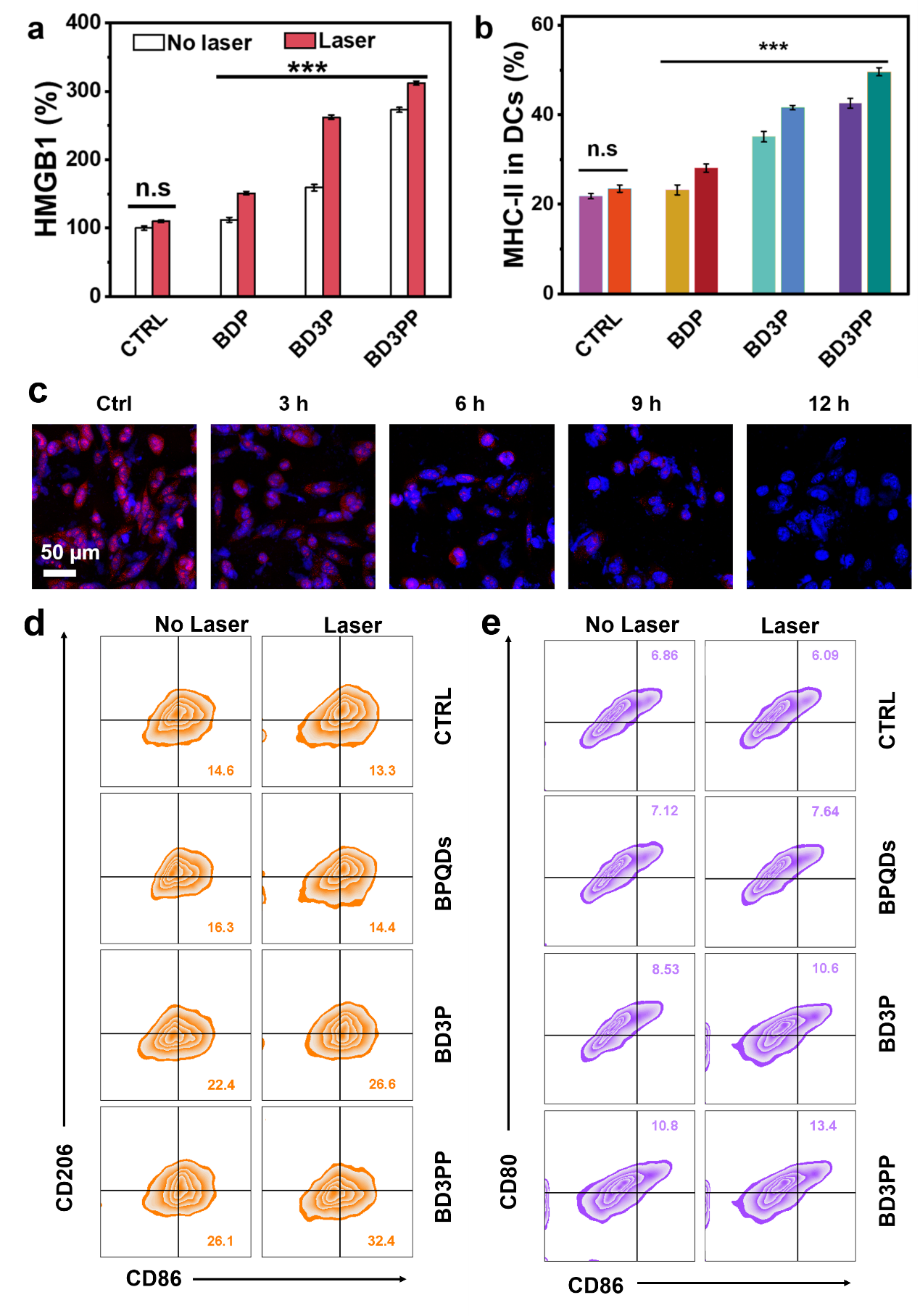


**Figure S14.** (a) Percentage of extracellular HMGB1 of 4T1 cells co-incubated with the material under laser stimulation. (b) Relative MHC-II expression on the surface of mature DCs. (c) Representative confocal fluorescence imaging of 4T1 cells from different times. HMGB1: red fluorescence; nucleus: blue fluorescence. (d) Representative FCAS of macrophage differentiation into M1 macrophages (CD86^+^CD206^−^). (e) Representative FCAS of co-stimulatory molecules CD80^+^CD86^+^ expressed on the surface of mature DCs and corresponding quantifications.


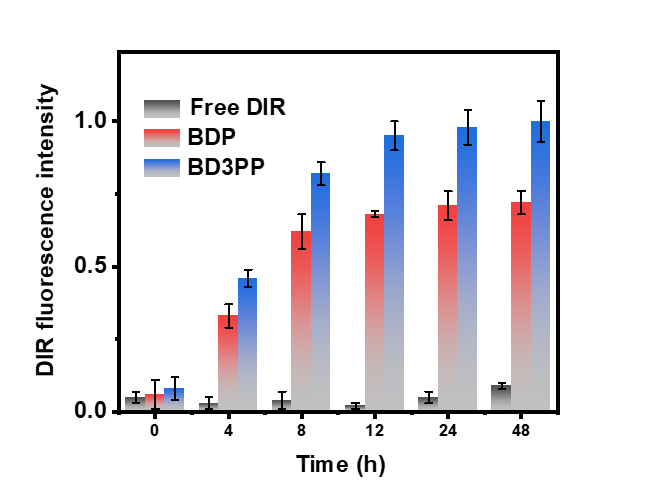


**Figure S15.** *Ex vivo* optical images of tissues for fluorescent NPs.


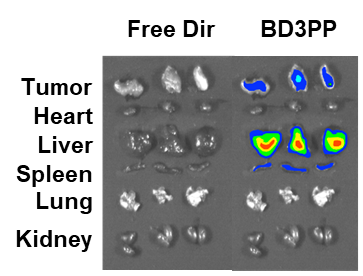


**Figure S16.** Imaging of isolated organs from mice under different treatments.


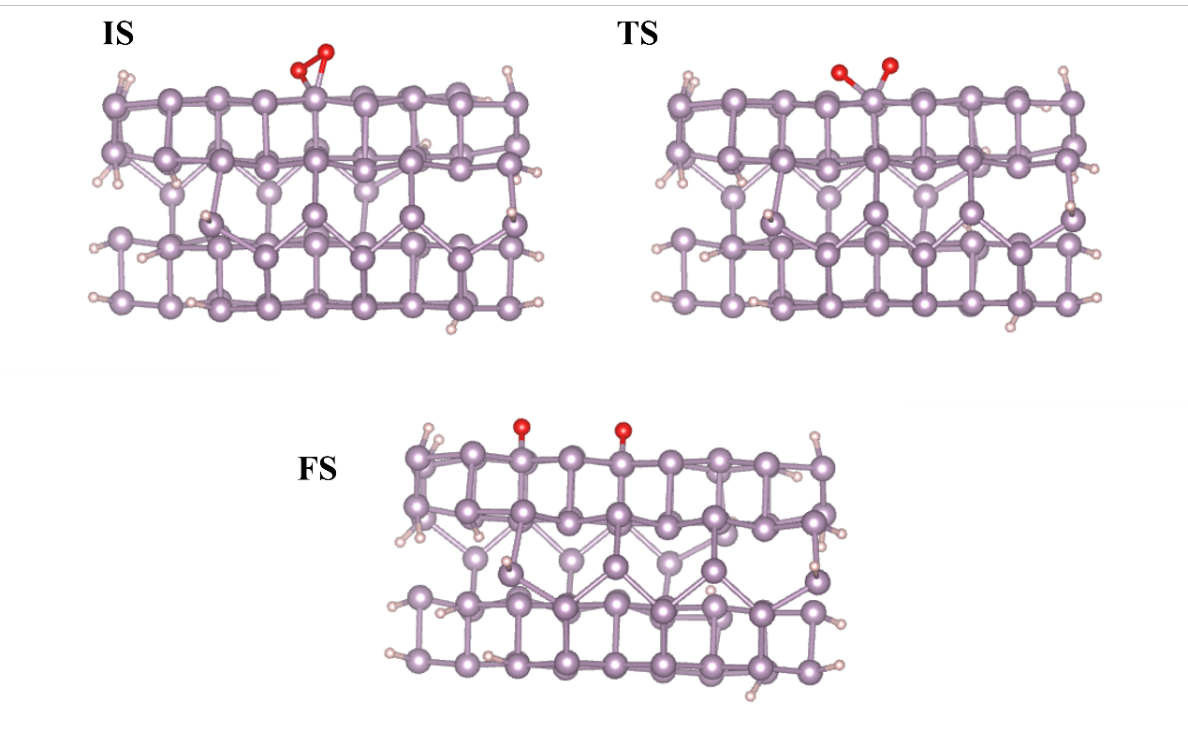


**Figure S17.** Snapshots of the regional configurations of the initial state (IS), transition state (TS), and final state (FS), produced by minimum energy pathway (MEP) calculations of O_2_ dissociation on the perfect surface of bilayer BPQDs.


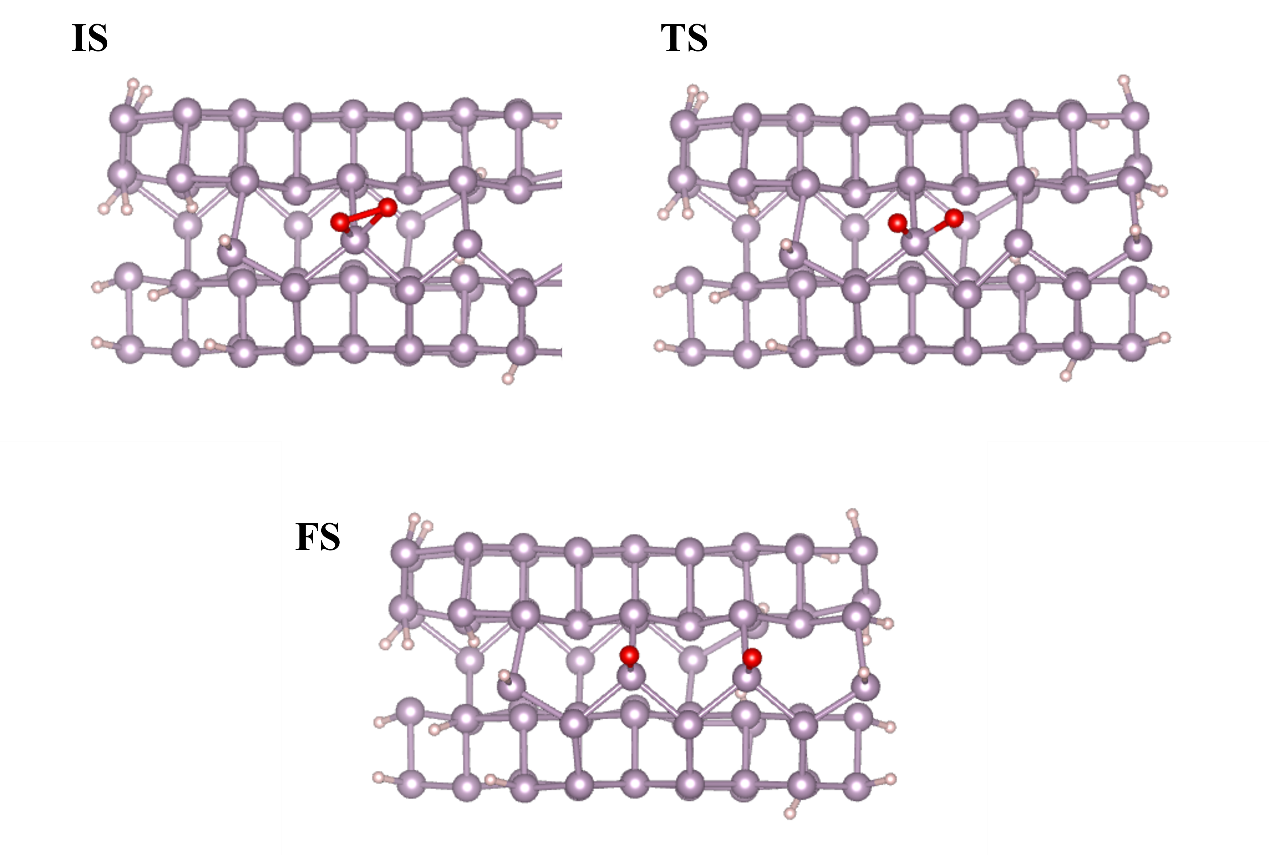


**Figure S18.** Snapshots of the regional configurations of the IS, TS, and FS, produced by MEP calculations of O_2_ dissociation on the perfect surface of bilayer BPQDs.


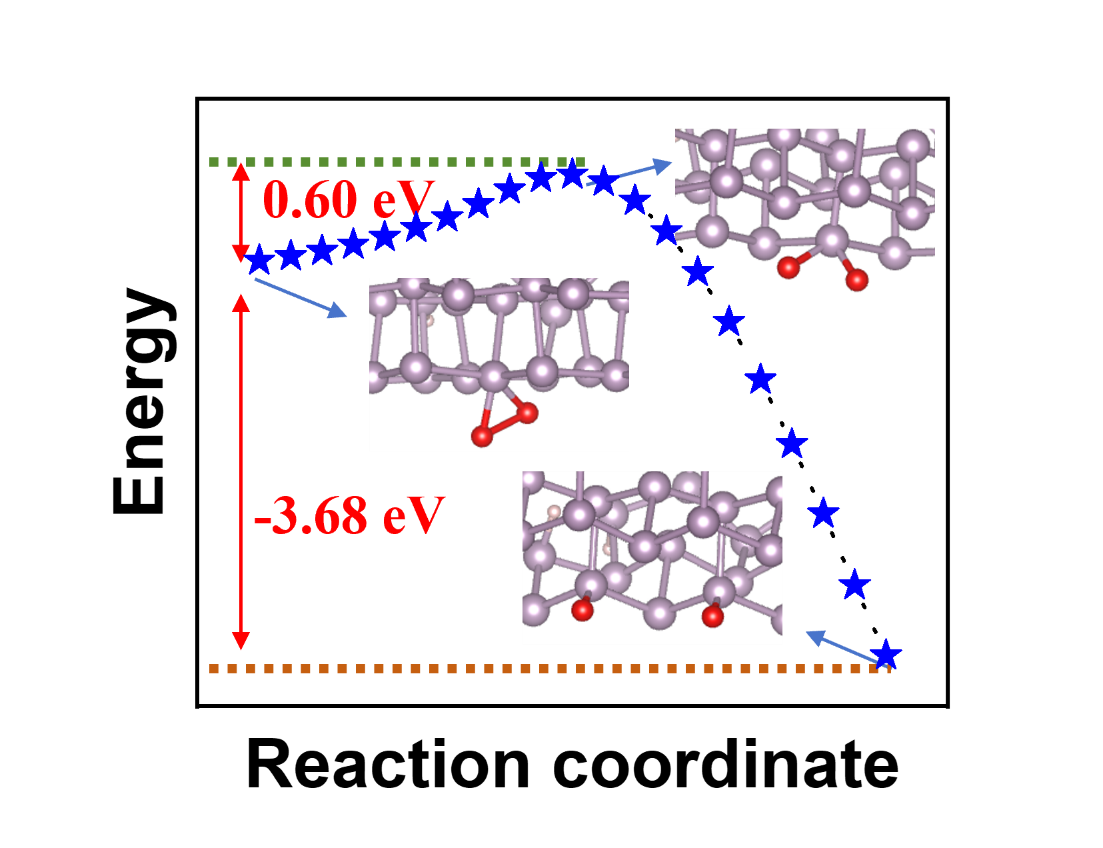


**Figure S19.** MEP calculations of O_2_ dissociation at the self-passivated zigzag edge of bilayer BPQDs.


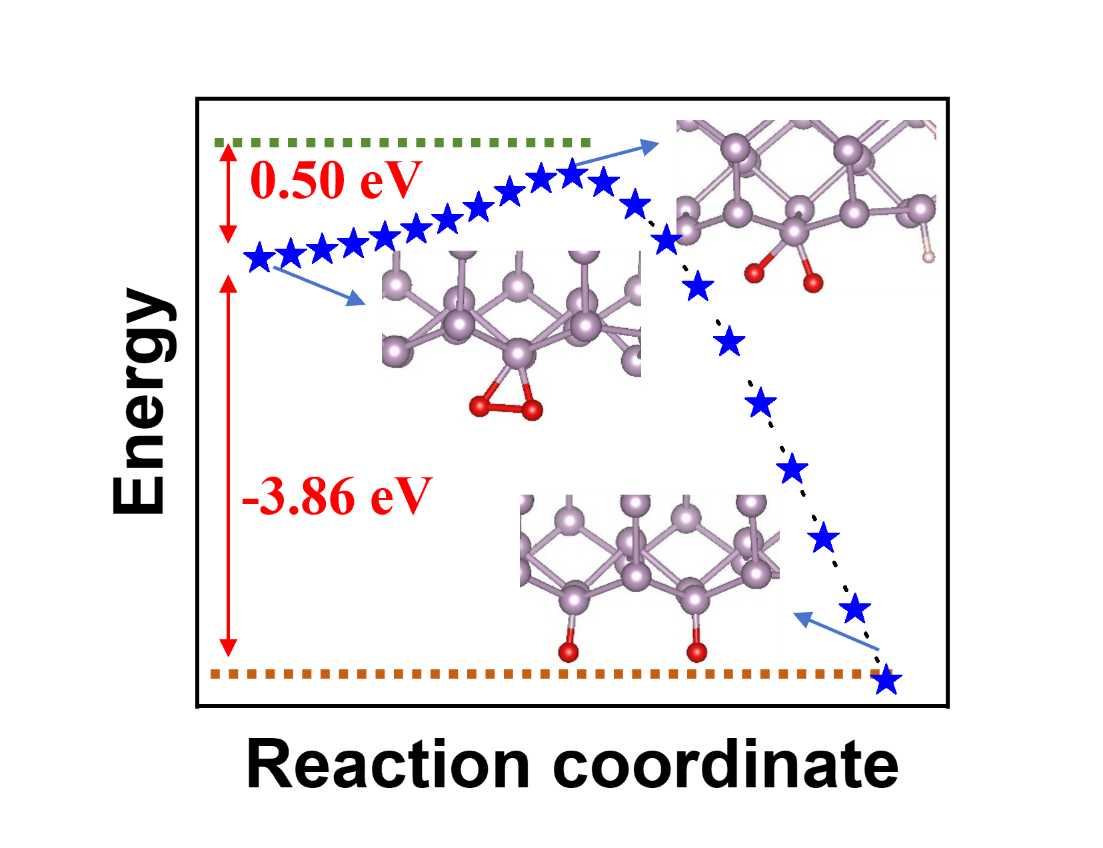


**Figure S20.** MEP calculations of O_2_ dissociation at the self-passivated zigzag edge of bilayer BPQDs.


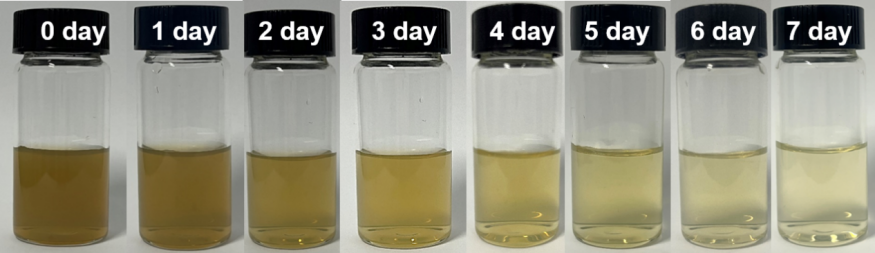


**Figure S21.** Photographs of BPQDs after storage in water for 7 days.

**Figure S22.** ζ-potential of BPQDs after storage in water for 7 days.


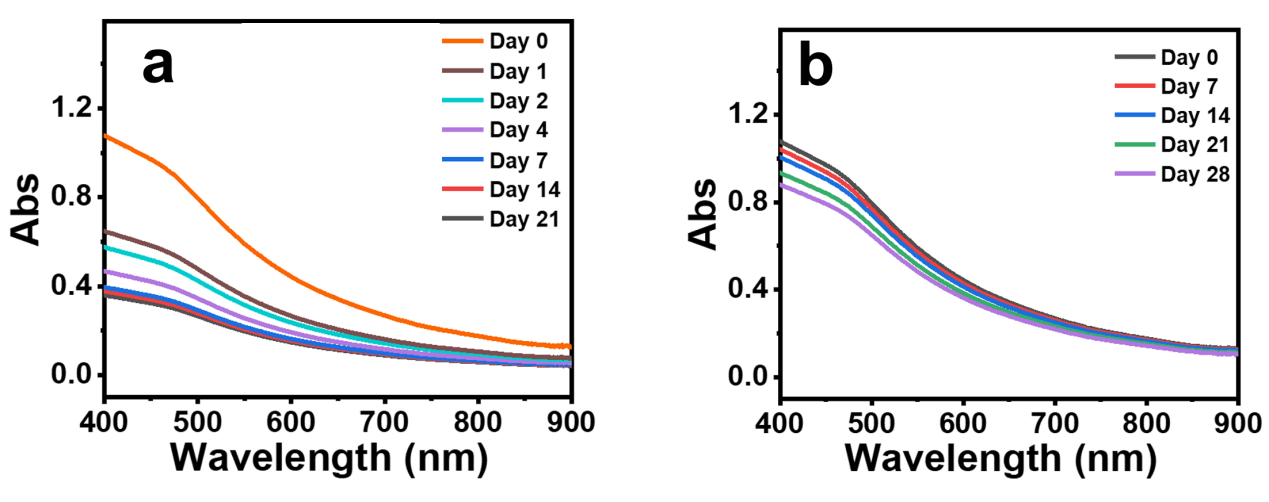


**Figure S23.** UV-vis spectrum of (a) BPQDs and (b) BPQDs@PLGA.

**Figure S24.** Photothermal heating curves of BPQDs and BD3PP after storage in water for different periods and irradiation with an 808 nm laser (1.0 W cm^-2^) for 10 min.

**
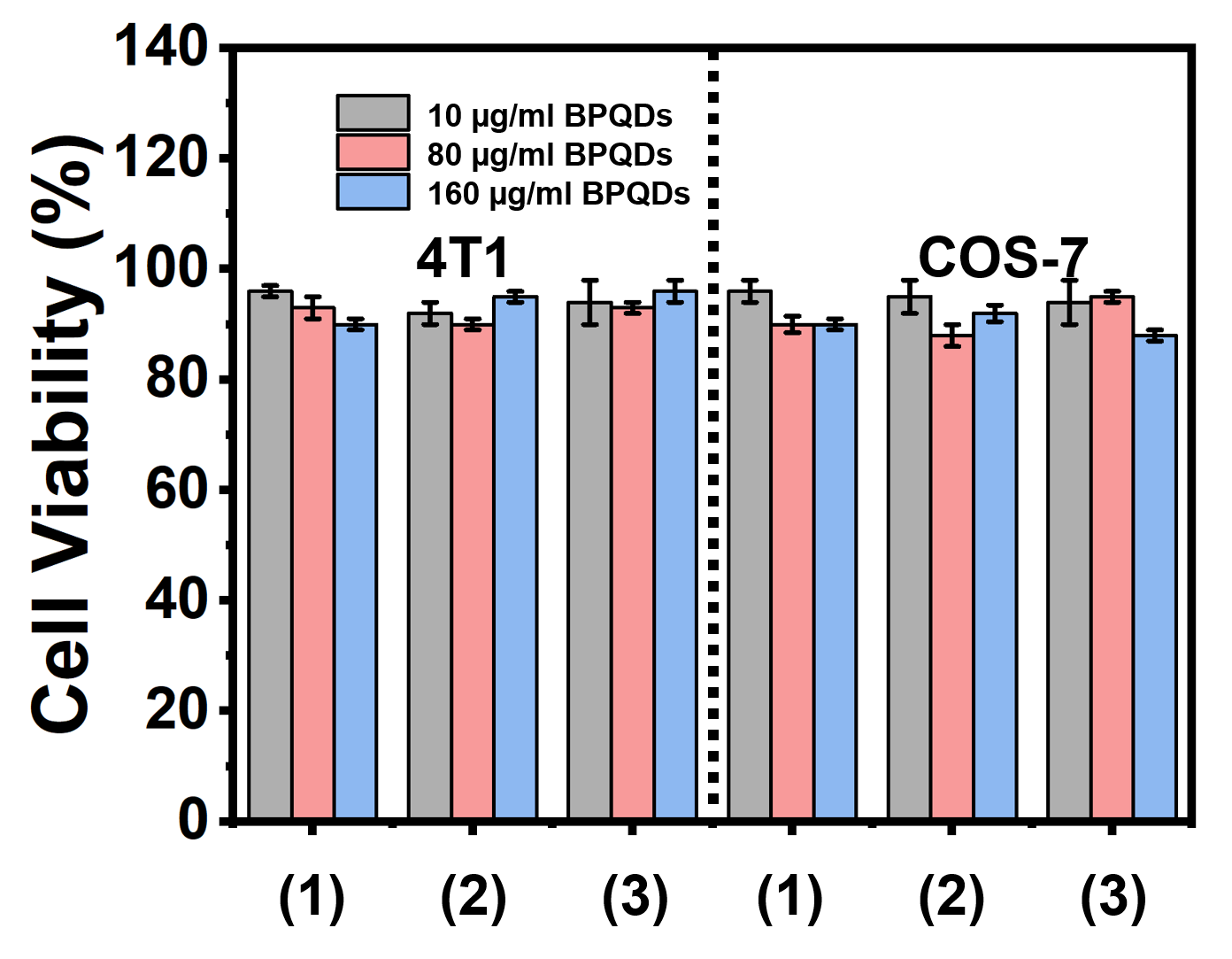
**

**Figure S25.** Cell viability of normal COS-7 cells and tumor 4T1 cells after incubation with newly prepared or 7-day- stored BPQDs for 24 h. (1) Control; (2) Newly prepared BPQDs; (3) BPQDs stored for 7 days.


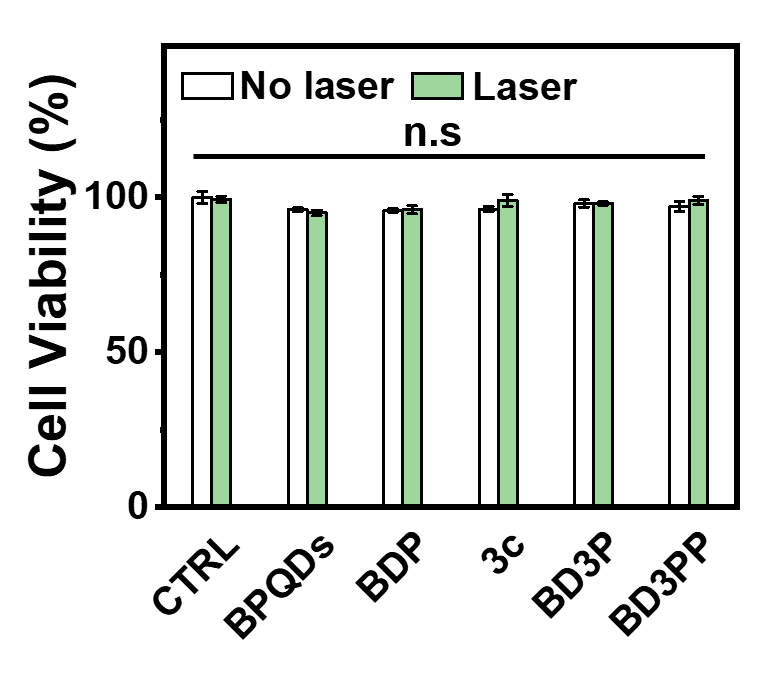


**Figure S26.** Cell viability of normal COS-7 cells after incubation with different groups for 48 h.

**
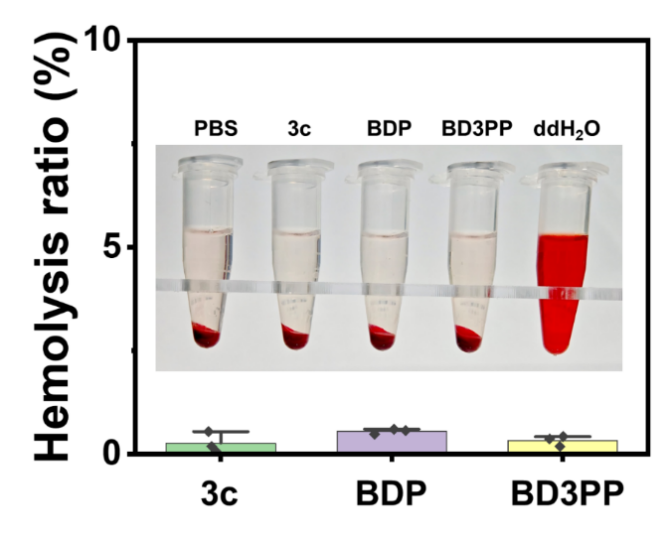
**

**Figure S27.** Hemolysis experiments of 3c, BDP, and BD3PP. Inset: photographs of tubes from the hemolysis experiments.


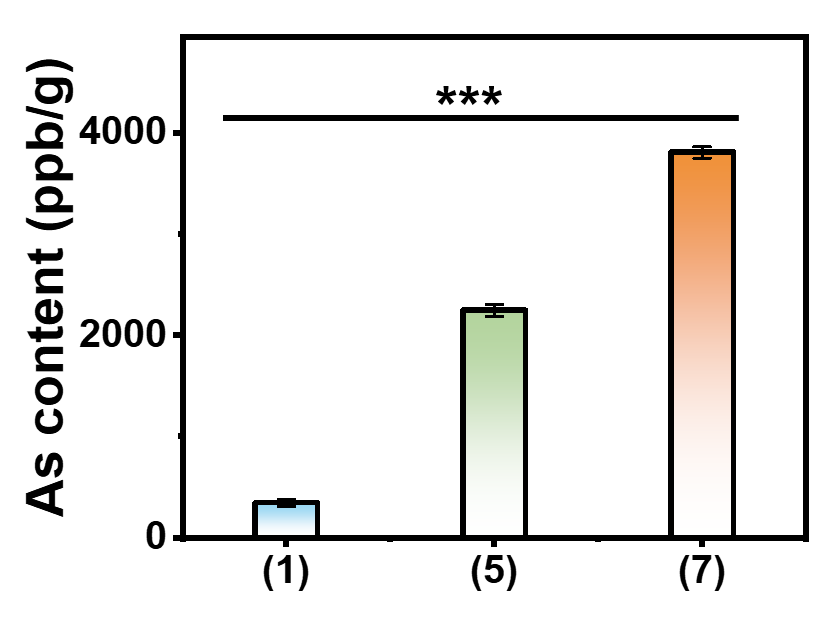


**Figure S28.** Intratumoral As content in different treatment groups.


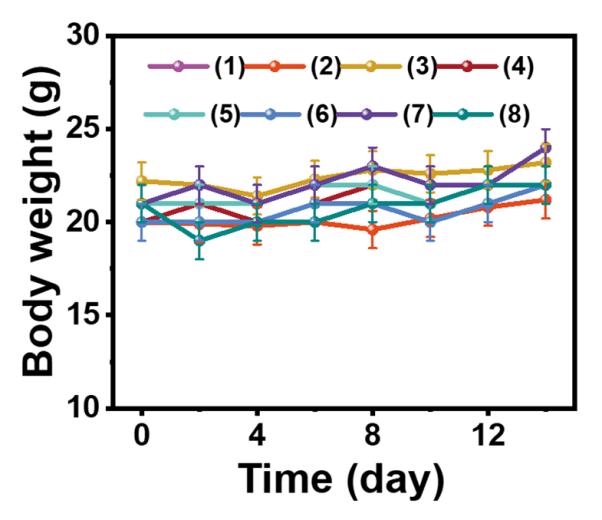


**Figure S29.** Body weight of mice over 14 days in different treatment groups.


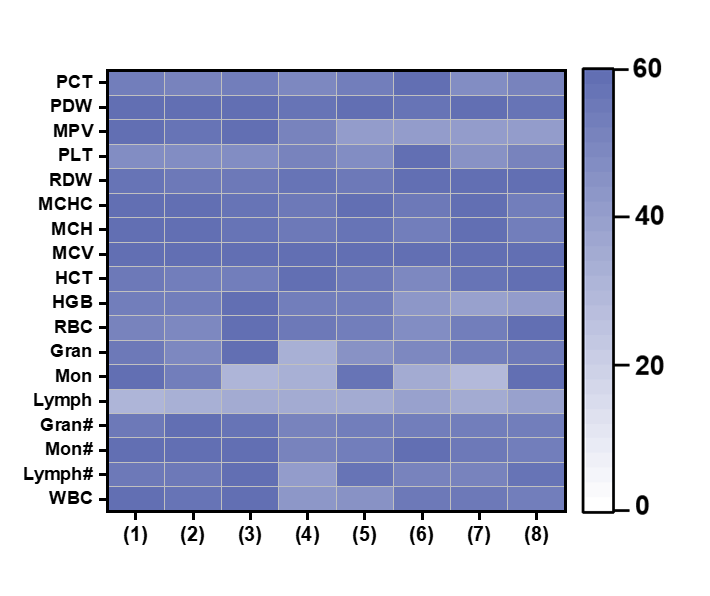


**Figure S30.** Routine blood analysis of 4T1 tumor-bearing mice in different treatment groups on day 14.


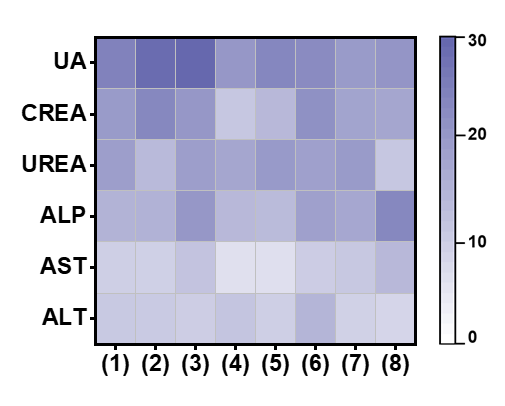


**Figure S31.** Liver and kidney function analysis of 4T1 tumor-bearing mice in different treatment groups on day 14.


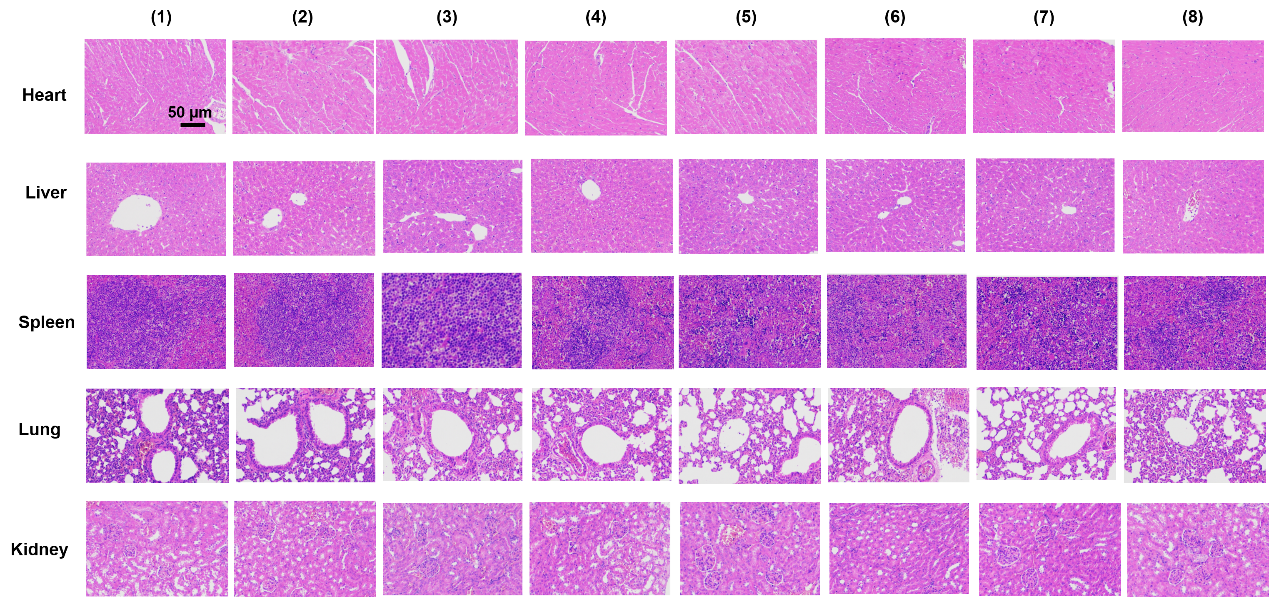


**Figure S32.** H&E staining of heart, liver, spleen, lung, and kidney. scale bar: 50 μm.

**Figure S33.** Survival rate of mice from these groups. (1): Control; (2): NIR; (3): BDP; (4): BDP+NIR; (5): BD3P; (6): BD3P+NIR; (7): BD3PP; (8): BD3PP+NIR.

**Table S1.** Drug loading efficiency and encapsulation efficiency determined by ICP analysis.

|  | Sample 1 | Sample 2 | Sample 3 | mean±SD |
| --- | --- | --- | --- | --- |
| Dry BD3PP weight/mg | 5.8 | 7.6 | 7.4 |  |
| As weight/mg | 0.754 | 0.765 | 0.841 |  |
| Loading efficiency/% | 13.0 | 10.1 | 11.4 | 11.5±1.4 |
| Encapsulation efficiency/% | 75.4 | 76.5 | 84.1 | 78.7±4.7 |
